# Pyrolysis‐Free Formed Cooperative Coupling Pt‐Co Dual‐Functional Sites for Saline Electrolysis with High Adaptability

**DOI:** 10.1002/advs.202509394

**Published:** 2025-10-07

**Authors:** Jianyue Yan, Wen‐Yan Li, Zhao‐Di Wang, Jian‐Hua Qin, Peng Peng, Shuang‐Quan Zang

**Affiliations:** ^1^ Henan Key Laboratory of Crystalline Molecular Functional Materials College of Chemistry Zhengzhou University Zhengzhou 450001 P. R. China; ^2^ College of Chemistry and Chemical Engineering Luoyang Normal University Luoyang 471934 P. R. China

**Keywords:** cooperative coupling, high adaptability, overall saline electrolysis, pyrolysis‐free

## Abstract

The development of large‐scale and low‐cost hydrogen production based on water splitting necessitates the design of well‐defined and highly adaptable catalytic materials. Advancements in synthesis strategies are crucial for the preparation of efficient catalysts and the understanding of underlying mechanisms. Here, a pyrolysis‐free strategy is present toward the directive assembly of cooperative coupling single cobalt atoms and fully exposed platinum nanoclusters as active sites. The catalysts possess dual functionality and high adaptability for overall saline electrolysis. As expected, both the electrode and the symmetric cell deliver good catalytic activity and stability. Additionally, the rational and controllable synthesis leads to comprehensive analysis, which reveals that the cooperative coupling between Pt‐Co sites can regulate the interaction between the intermediates and active centers to improve the activity, prevent the Cl^−^ poisoning, and ensure the efficient selectivity. The catalytic performance has been well‐maintained in not only saline water, but also fluctuant and extreme concentrations of hydroxide. This work expedites the research progress and future deployment of rationally designed and efficient saline or seawater electrolyzers.

## Graphical abstract

Through a mild pyrolysis‐free route, cooperative coupling of single cobalt atoms (CoSA) and fully exposed platinum nanoclusters (PtNC) is assembled. As expected, the as‐prepared catalysts PtNC/CPF‐Co exhibit dual‐functional performance and high adaptability, demonstrating low overpotentials of 44 mV for HER and 340 mV for OER at 10 mA cm^−2^ in saline electrolysis.

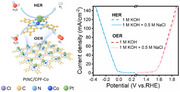

## 1 Introduction

Hydrogen boasts a considerably high energy density and serves as the feedstock for versatile industrial applications. Hydrogen energy, as a clean and efficient energy carrier, represents a paradigm shift in addressing climate change and energy security, holding transformative potential for global energy systems and environmental sustainability.^[^ ^1^ ^]^ The unique combination of hydrogen energy, containing low emissions, high energy density, and adaptability across sectors, positions itself as a cornerstone of the future energy landscape.^[^ ^1^, ^2^, ^3^ ^]^ Notably, the synergistic approach to developing hydrogen as a medium for energy storage and conversion holds the capability to integrate renewable energy into the power system while addressing its inherent intermittency and variability.^[^ ^4^ ^]^ Such bidirectional energy conversion and dynamic balance between electricity and hydrogen could offer a scalable solution to the intermittency of renewables, ensuring the grid stability and flexibility while maximizing renewable utilization.^[^ ^5^, ^6^, ^7^ ^]^

Recent breakthroughs in hydrogen production and storage technologies have accelerated the feasibility.^[^ ^8^, ^9^, ^10^, ^11^, ^12^ ^]^ Innovations of water electrolysis powered by renewables foster the research and infrastructure development of hydrogen production. Specifically, saline water, including seawater, which covers over 70% of the Earth's surface, offers a nearly limitless and sustainable feedstock for hydrogen generation while addressing the scarcity of freshwater resources.^[^ ^13^, ^14^, ^15^ ^]^ Nevertheless, the production costs for green hydrogen remain high because the direct utilization of saline water faces critical challenges such as severe chlorine evolution reactions and catalyst degradation.^[^ ^16^ ^]^ Additionally, the reliance on carbon‐based materials for electrocatalysts introduces issues such as structural instability under harsh electrolytic conditions and environmental concerns, especially under the high concentration alkali solutions (commonly 30% by weight) during alkaline water electrolysis. Since catalysts are pivotal in water electrolysis, these formidable challenges necessitate the development of highly stable and efficient electrocatalysts that can operate in the widely used industrial environments.^[^ ^17^, ^18^, ^19^ ^]^

In most cases, during saline electrolysis, chloride (Cl^−^) corrosion could result in catalyst deactivation via Cl^−^ adsorption and oxidation, while the high concentration and fluctuation of hydroxide (OH^−^) would undermine the hydrogen evolution reaction (HER) efficiencies.^[^ ^15^, ^16^, ^20^ ^]^ Thus, traditional Pt‐based catalysts exhibit optimal HER activity but suffer from rapid deactivation. For the oxygen evolution reaction (OER), IrO_2_ and RuO_2_ are benchmark catalysts, yet their high cost and poor stability in alkaline solutions, especially saline water, hinder their practical applications. By far, tremendous efforts have been devoted to addressing the observed bottleneck.^[^ ^21^, ^22^, ^23^, ^24^, ^25^ ^]^ Recent seminal works have reported advanced catalysts such as the CuMo_6_S_8_/Cu and Pt/NiMo electrodes, which demonstrated stable hydrogen evolution even at ampere‐level current density.^[^ ^26^, ^27^ ^]^ Aiming at the corrosion arising from the adsorption of Cl^−^, the negatively charged anionic layer upon oxidation and induced chlorine‐repelling effect was developed to protect the anode.^[^ ^28^ ^]^ For example, the NiFe LDH/NiSx/Ni foam and NiMoN@NiFeN electrode could stably operate in NaCl electrolyte.^[^ ^9^, ^31^ ^]^ Catalysts with non‐precious metal and synergistic effects hold the promising potential toward minimized cost, promising activity, and high environmental adaptability.^[^ ^32^, ^33^, ^34^, ^35^, ^36^, ^37^ ^]^

Meanwhile, challenges also exist in the system assembly and scaled‐up synthesis of catalysts. To this point, dual‐functional catalysts that simultaneously drive reduction and oxidation exhibit distinct advantages, particularly in complex environments like saline.^[^ ^38^ ^]^ The heterointerfaces and compositional synergies of dual‐functional catalysts could be leveraged to significantly reduce the reliance on noble metal while optimizing electron transfer pathways.^[^ ^39^, ^40^, ^41^, ^42^, ^43^, ^44^, ^45^ ^]^ For instance, the CPF‐Ni/Fe reported by our group and Ru(x)SACs@FeCo‐LDH outperformed the monofunctional Pt and IrO_2_‐based systems with reduced reaction overpotentials for both HER and OER.^[^ ^46^, ^47^ ^]^ Besides, serving as dual electrodes could eliminate cross‐contamination and interface mismatch issues, thus simplifying the assembly and improving the stability. Along with the challenges of cathode OH^−^ fluctuation and anode Cl^−^ corrosion in continuous feeding and electrolysis under industrial processes, it is vital to develop new synthetic strategies of multi‐functional catalysts toward high activity, cost‐efficiency, long‐term stability, and large‐scale synthesis.

In this work, we have assembled the cooperative coupling of single cobalt atoms (CoSA) and fully exposed platinum nanoclusters (PtNC) via a mild pyrolysis‐free route. Such dual‐sites not only ensure the geometric confinement and stability, but also activate unconventional dual‐catalytic function through electronic modulation: PtNCs optimize HER activity via facile H⁺ adsorption, while CoSAs facilitate OER inhibition through highly‐selective OH^−^ adsorption/electron transfer pathways. As expected, the as‐prepared catalysts (PtNC/CPF‐Co) exhibit dual‐functional performance in 1.0 m KOH + 0.5 m NaCl electrolytes, demonstrating low overpotentials of 44 mV for HER and 340 mV for OER at 10 mA cm^−2^. Impressively, except for the tolerance to Cl^−^ corrosion, PtNC/CPF‐Co could also maintain the electrocatalytic performance in varying concentrations and even concentrated KOH electrolyte, possessing considerable environmental adaptability. Furthermore, our synthetic strategy engineers the cooperative coupling catalytic sites with precisely controlled electronic configurations and spatial distributions, providing rich opportunities for theoretical insights. Hence, the charge transfer dynamics could be thoroughly elucidated by density functional theory (DFT) calculations. The as‐induced electron redistribution could enhance overall catalytic efficiency while suppressing Cl^−^ adsorption, enabling efficient water‐splitting kinetics and exceptional tolerance. Moreover, the pyrolysis‐free synthesis enables the scale‐up process while eliminating normal energy‐intensive pyrolysis steps, positioning our material as a promising candidate for efficient saline or seawater electrolyzers.

## 2 Results and Discussion

### 2.1 Synthesis and Characterization

**Figure 1:**
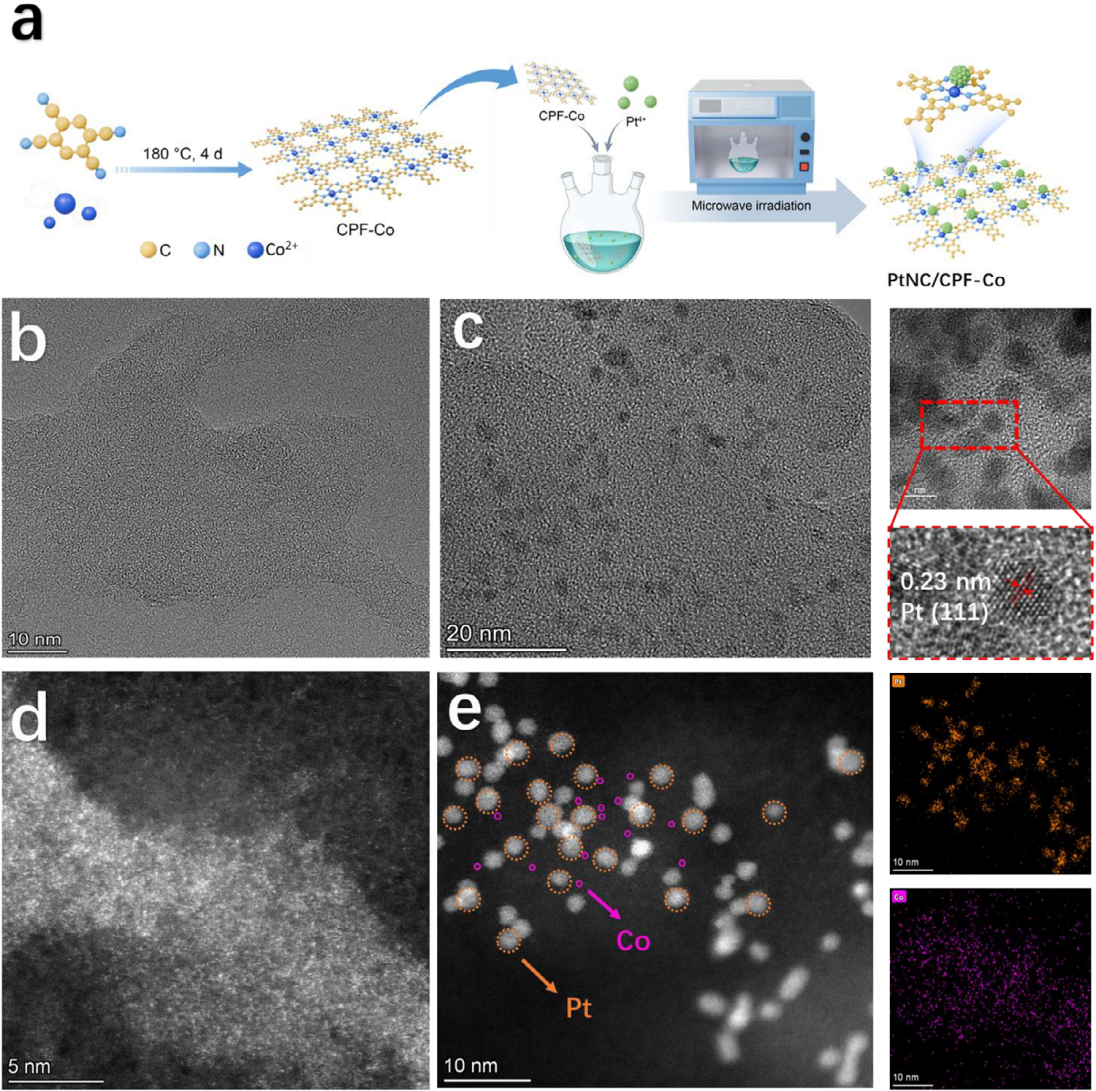
a) Schematic illustration of the synthesis of CPF‐Co and followed PtNC/CPF‐Co. b,c) Transmission electron microscopy (TEM) images of CPF‐Co and PtNC/CPF‐Co. Right: High‐resolution transmission electron microscopy (HRTEM) image of PtNC/CPF‐Co. d) and e) HAADF STEM images of CPF‐Co and PtNC/CPF‐Co. Right: EDS elemental mapping images of Co atoms and Pt clusters of PtNC/CPF‐Co.

The conjugated phthalocyanine frameworks of CPF‐Co were formed via pyrolysis‐free synthesis. The benzene‐1,2,4,5‐tetracarbonitrile (BTC) was used as the building block with cobalt as the centred metal and finally connected into a confined network containing extended π conjugation. Subsequently, the fully‐exposed Pt clusters were riveted on the frameworks through in situ reduction of platinum chloride under mild microwave conditions (**Figure** **1**a). The peaks at ≈1547, 1697, 1711, and 1757 cm^−1^ in Fourier transform infrared spectroscopy (FT‐IR) (Figure S1, Supporting Information) suggested the as‐designed formation of the macrocyclic structure of phthalocyanine in both CPF‐Co and composite PtNC/CPF‐Co. As compared in the powder X‐ray diffraction (PXRD) patterns (Figure S2, Supporting Information), the additional peaks of PtNC/CPF‐Co at 39.5°, 46.4°, and 67.1° indicated the (111), (200), and (220) planes of face‐centered cubic Pt, confirming the successful incorporation of Pt clusters.^[^ ^48^ ^]^ Meanwhile, as displayed in the transmission electron microscopy (TEM) images, raw CPF‐Co possessed 2D layered structures without any aggregates (Figure 1b; Figure S3, Supporting Information), while uniformly distributed nanoclusters could be found along the PtNC/CPF‐Co with a homogeneous diameter of ≈2 nm (Figure 1c). Notably, under the high‐angle annular dark‐field scanning transmission electron microscope (HAADF‐STEM), abundant single Co atoms could be found in the CPF‐Co (Figure 1d). As for PtNC/CPF‐Co, Pt nanoclusters and single‐atomic Co sites concordantly existed in the HAADF‐STEM images (Figure 1e).^[^ ^49^ ^]^ The corresponding elemental mappings clearly showcased the homogeneous distribution of C, N, Co, and Pt (Figure 1e; Figures S4 and S5, Supporting Information).

**Figure 2:**
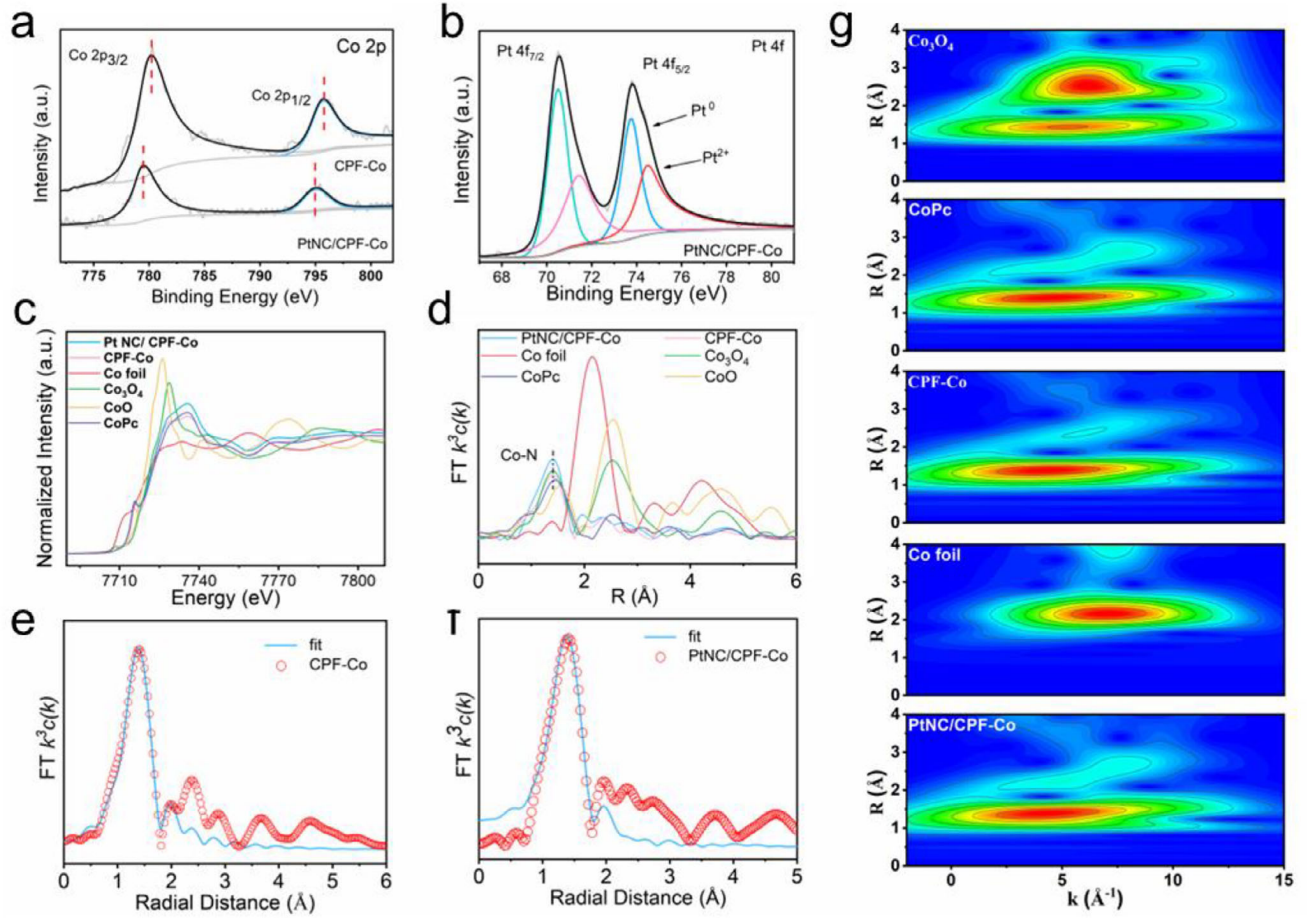
a) Co 2p spectra of PtNC/CPF‐Co and CPF‐Co. b) Pt 4f spectra of PtNC/CPF‐Co. c) X‐ray absorption near‐edge structure (XANES) and d) extended X‐ray absorption fine structure (EXAFS) spectra of Co K‐edge for PtNC/CPF‐Co, CPF‐Co, Co‐foil, CoPc, Co_3_O_4,_ and CoO. e) Quantitative EXAFS curve fitting in the R space of CPF‐Co. f) Quantitative EXAFS curve fitting in R space of PtNC/CPF‐Co. g) WT‐EXAFS of Co K‐edge for Co foil, Co_3_O_4_, CoPc, CPF‐Co, and PtNC/CPF‐Co.

Furthermore, the inherent chemical state of CPF‐Co and PtNC/CPF‐Co was thoroughly analyzed and deeply studied. According to the survey spectra of X‐ray photoelectron spectroscopy (XPS), each of the samples possessed as‐designed elemental information (Figures S6 and S7, Supporting Information). In the high‐resolution spectra, the Pt 4f spectrum of PtNC/CPF‐Co demonstrated the peaks at 70.5 and 73.8 eV, indicating to the Pt^0^ state, while a couple of peaks, 71.4 and 74.5 eV, arose from the surface oxidized state of Pt^2+^ (**Figure** **2**b).^[^ ^50^ ^]^ Meanwhile, the high‐resolution Co 2p spectra of CPF‐Co displayed the couple peaks at 780.3 and 795.8 eV, which were shifted in PtNC/CPF‐Co, indicating the electron transform tendency on central Co sites after cooperation with Pt nanoclusters (Figure 2a).^[^ ^51^, ^52^, ^53^ ^]^ Moreover, the synchrotron‐based X‐ray absorption near‐edge structure (XANES) and the extended X‐ray absorption fine structure (EXAFS) spectra were employed to get deep insight into the coordination status, bonding configurations, and the electronic interaction between PtNCs and CPF‐Co. As shown in Figure 2c,d, the EXAFS spectra of both CPF‐Co and PtNC/CPF‐Co demonstrated prominent peaks at ≈1.41 Å, attributing to the Co‐N coordination. None of the typical scattering peaks for Co‐Co (2.14 Å) or Co‐O‐Co (2.54 Å) coordination compared to Co foil and Co_3_O_4_, excluding the presence of metallic or oxidative cobalt nanoparticles. In the XANES analysis, the pre‐edge profile of CPF‐Co demonstrated similar signals to those of cobalt phthalocyanine (CoPc), which is attributed to the shakedown transition characteristic for square planar configuration. The XANES spectra of PtNC/CPF‐Co displayed a noticeable negative shift in the Co K‐edge compared with CPF‐Co, suggesting the cooperative coupling between Pt nanoclusters and Co centers.^[^ ^48^, ^54^ ^]^ According to these results, the calculated average coordination number (CN) of the Co‐N shell was ≈4, indicating a conventional single‐atom Co‐N4 structure (Tables S1 and S2, Supporting Information). Meanwhile, the Fourier transforms (FTs)‐EXAFS spectrum of the Co L3‐edge (Figure 2e,f; Figure S8, Supporting Information) revealed that compared with CPF‐Co, the EXAFS fitting results of PtNC/CPF‐Co indicated a similar Co─Co length of the first shell but with a higher coordination number. The above bonding information and coordination structures were then further confirmed by the wavelet‐transform (WT) EXAFS spectra (Figure 2g).

### 2.2 Catalytic and Adaptive Performance

**Figure 3:**
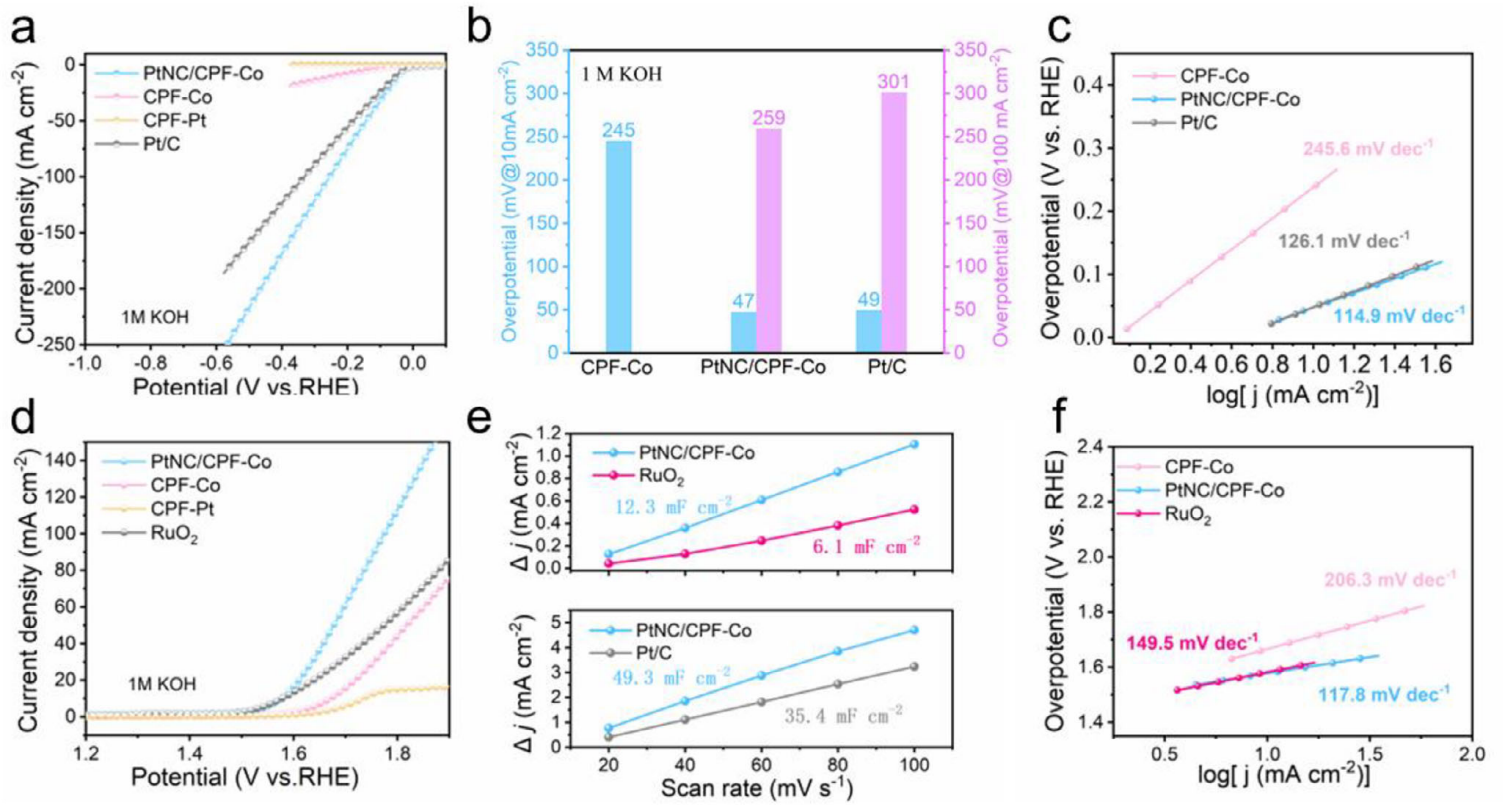
HER performance: a) LSV curves, b) overpotential, and c) corresponding Tafel slopes of PtNC/CPF‐Co, CPF‐Co, CPF‐Pt, and commercial Pt/C catalysts in 1 m KOH media. OER performance: d) LSV curves and f) corresponding Tafel slopes of PtNC/CPF‐Co, CPF‐Co, CPF‐Pt, and commercial RuO_2_ catalysts in 1 m KOH media. e) ECSA of PtNC/CPF‐Co and RuO_2_ for the OER process (up); ECSA of PtNC/CPF‐Co and Pt/C for the HER process.

The dual‐functional catalytic performance of the as‐synthesized CPF‐Co, CPF‐Pt, and PtNC/CPF‐Co was evaluated under a standard three‐electrode system. In a conventional alkaline electrolyte (1 m KOH), PtNC/CPF‐Co exhibited superior activity for HER to CPF‐Co and CPF‐Pt with the overpotentials of 47 mV at 10 mA cm^−2^ during linear sweep voltammetry (LSV), which was comparable with commercial Pt/C (49 mV) under the same conditions (**Figure** **3**a,b). The Tafel slope of PtNC/CPF‐Co (114.9 mV dec^−1^) was much smaller than that of CPF‐Co (245.6 mV dec^−1^) and similar to Pt/C (126.1 mV dec^−1^), indicating the proper reaction kinetics (Figure 3c). Notably, the cooperative coupling Pt‐Co active sites also possessed considerable catalytic ability for OER. In the same electrolyte, the overpotentials at 10 mA cm^−2^ of CPF‐Co, CPF‐Pt, PtNC/CPF‐Co, and RuO_2_ were 440, 505, 340, and 340 mV, respectively (Figure 3d). The Tafel slope of PtNC/CPF‐Co for OER was only (117.8 mV dec^−1^), which was even lower than that of RuO_2_ (149.5 mV dec^−1^) (Figure 3f).^[^ ^55^, ^56^ ^]^ Moreover, in the electrochemical impedance spectroscopy (EIS), PtNC/CPF‐Co demonstrated the lowest charge transfer resistance (*R_ct_*) among the prepared catalysts (Figure S9, Supporting Information), ensuring the enhanced catalytic activity.^[^ ^57^ ^]^ As shown in the electrochemically active surface area (ECSA) based on the double‐layer capacitance (*C_dl_*) (Figure 3e; Figures S10 and S11, Supporting Information), during the HER process, the value of PtNC/CPF‐Co (42.3 mF cm^−2^) was higher than the value of commercial Pt/C (35.4 mF cm^−2^). For OER catalysis, the value of PtNC/CPF‐Co (12.3 mF cm^−2^) was even two times higher than the value of RuO_2_ (6.1 mF cm^−2^), indicating the significant promotion in the density of active sites. Thus, it could be concluded that the as‐designed PtNC/CPF‐Co contained comparable catalytic activity with Pt/C for HER, and similar catalytic activity with RuO_2_ for OER, suggesting promising application potentials.

**Figure 4:**
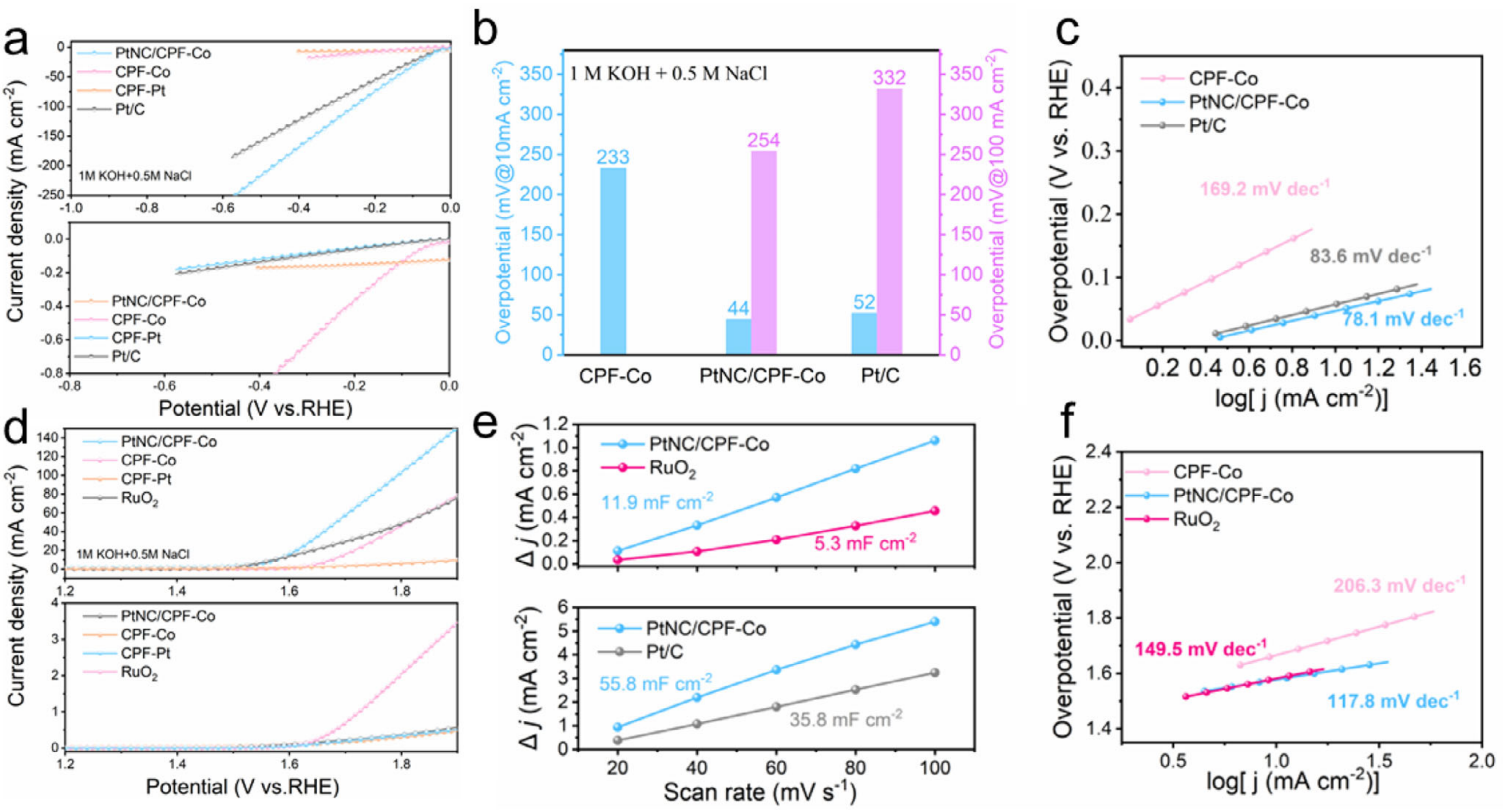
HER performance: a) LSV curves (up), LSV curves normalized to the ECSA (down), b) overpotential, and c) corresponding Tafel slopes of PtNC/CPF‐Co, CPF‐Co, and commercial Pt/C catalysts in 1 m KOH + 0.5 m NaCl media. OER performance: d) LSV curves (up), LSV curves normalized to the ECSA (down), and f) corresponding Tafel slopes of PtNC/CPF‐Co, CPF‐Co, CPF‐Pt, and commercial RuO_2_ catalysts in 1 m KOH + 0.5 m NaCl media. e) ECSA of PtNC/CPF‐Co and RuO_2_ for the OER process (up); ECSA of PtNC/CPF‐Co and Pt/C for the HER process (down).

Except for the dual‐functional catalytic activity, the cooperative coupling Pt‐Co sites also generated impressive environmental adaptability. With the same three‐electrode configuration utilizing alkaline seawater electrolyte (1 m KOH + 0.5 m NaCl), the catalytic performance for both HER and OER was further evaluated. As displayed in **Figure** **4**, the activity for HER was well reserved. The overpotentials of PtNC/CPF‐Co from LSV were quite comparable with Pt/C at 10 and 100 mA cm^−2^ (Figure 4a,b), while the Tafel slope of PtNC/CPF‐Co (78.1 mV dec^−1^) was even superior to Pt/C (83.6 mV dec^−1^) (Figure 4c). PtNC/CPF‐Co still held the lowest charge transfer resistance compared with CPF‐Co and Pt/C, ensuring the catalytic activity (Figure S12, Supporting Information). Moreover, the cooperative coupling sites generated efficient selectivity for oxidation reaction (vide infra), leading to stable catalytic performance during OER. The LSV curve of PtNC/CPF‐Co was quite maintained after the addition of sodium chloride, with a consistent low Tafel slope (Figure 4d,f). Meanwhile, during the HER process, the *C_dl_* value of PtNC/CPF‐Co (55.8 mF cm^−2^) was still higher than Pt/C (35.8 mF cm^−2^) (Figure 4e; Figure S13, Supporting Information). For OER, the *C_dl_* value of PtNC/CPF‐Co (11.9 mF cm^−2^) was even much higher than RuO_2_ (5.3 mF cm^−2^), suggesting the larger active surface area (Figure 4e; Figure S14, Supporting Information). Besides, as shown in the chronopotentiometry tests (Figure S15, Supporting Information), PtNC/CPF‐Co exhibited superior stability in a conventional alkaline electrolyte. In the alkaline seawater electrolyte, PtNC/CPF‐Co still performed well without significant loss and exhibited negligible overpotential variation compared with that in the conventional electrolyte. Meanwhile, based on the ion chromatography, none of ClO^−^ could be detected after the long‐term chronopotentiometry test, confirming the high selectivity of PtNC/CPF‐Co and the effective inhibition toward the chlorine evolution reaction. The TEM analysis of the catalysts after the stability tests (Figure S16, Supporting Information) proved the structure and morphology stability, further demonstrating the good environmental adaptability.

**Figure 5:**
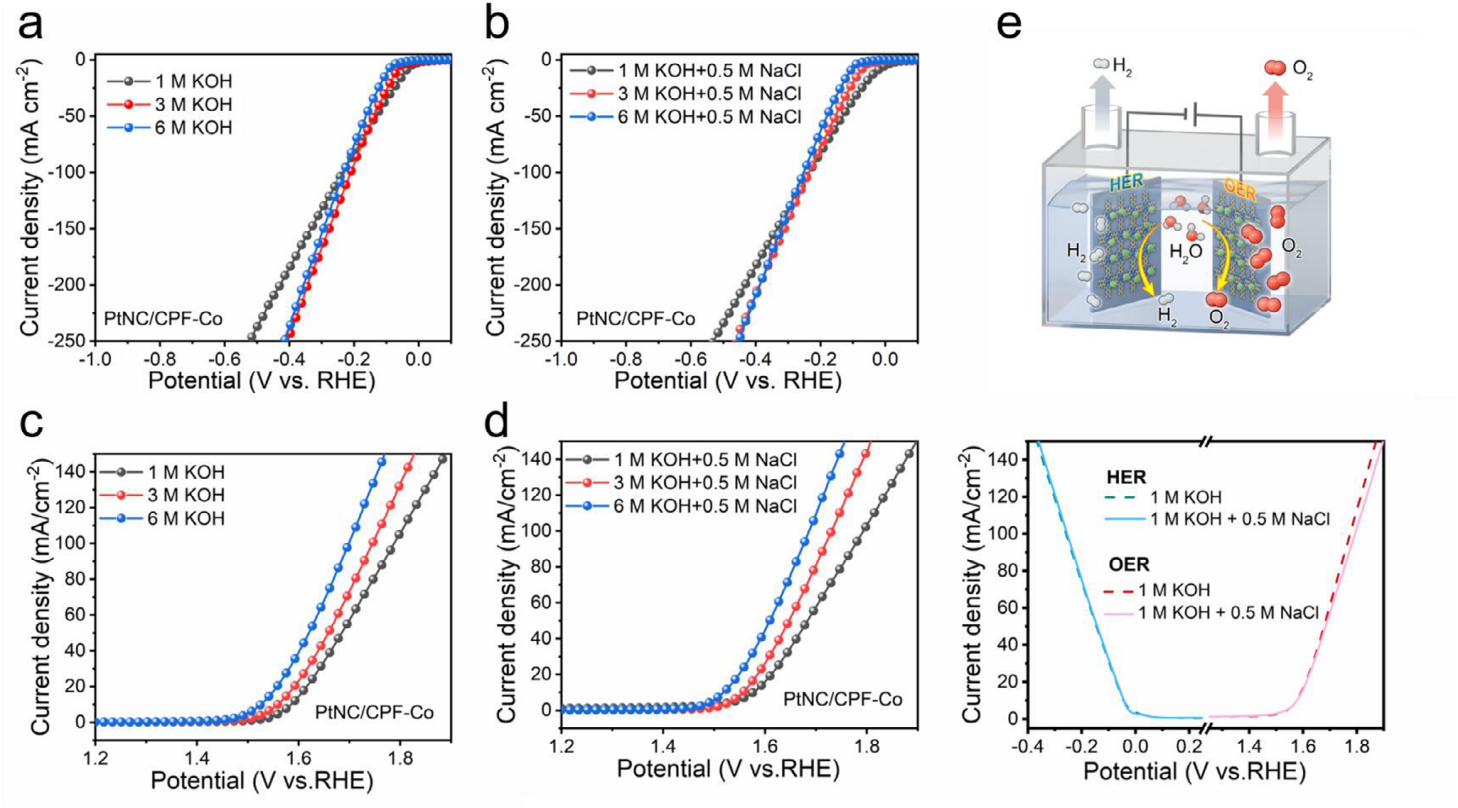
LSV curves of HER for PtNC/CPF‐Co in a) 1, 3, and 6 m KOH media and b) 1, 3, and 6 m KOH + 0.5 m NaCl media. LSV curves of for PtNC/CPF‐Co in c) 1, 3, and 6 m KOH media and d) 1, 3, and 6 m KOH + 0.5 m NaCl media. e) The overall water‐splitting performance of the symmetric PtNC/CPF‐Co||PtNC/CPF‐Co cell in 1 m KOH and 1 m KOH + 0.5 m NaCl.

Moreover, given to the industrial application requirements, the adaptability of the cooperative coupling Pt‐Co sites was verified in fluctuant and extreme pH conditions. As shown in **Figure** **5**a, even though the increase of hydroxide concentration would inevitably inhibit the HER process, the catalytic performance of PtNC/CPF‐Co for HER was only slightly affected when the KOH concentration increased from 1 to 6 m. For OER process, the performance was as expected enhanced along the increasing concentration of hydroxide, suggesting the promising activity of PtNC/CPF‐Co (Figure 5c). Impressively, the adaptability of PtNC/CPF‐Co was well remained with the addition of sodium chloride. As shown in Figure 5b,d, with the increase of KOH concentration in the electrolyte containing 0.5 m NaCl, the catalytic performance of PtNC/CPF‐Co demonstrated negligible differences compared with the homologous alkaline electrolyte. Furthermore, the overall water‐splitting performance of the dual‐functional PtNC/CPF‐Co was also explored in a two‐electrode cell to assess the practical feasibility (Figure 5e). In a conventional alkaline electrolyte (1 m KOH), the symmetric PtNC/CPF‐Co||PtNC/CPF‐Co cell achieved the overall water splitting voltage of 1.62 V at 10 mA cm^−2^. Impressively, in alkaline seawater electrolyte (1 m KOH + 0.5 m NaCl), the performance of symmetric PtNC/CPF‐Co||PtNC/CPF‐Co was well reserved. The symmetric cell demonstrated considerable stability no matter with or without the addition of sodium chloride (Figure S17, Supporting Information), confirming the remarkable resistance ability to harsh environments.

### 2.3 Mechanism Insights

**Figure 6:**
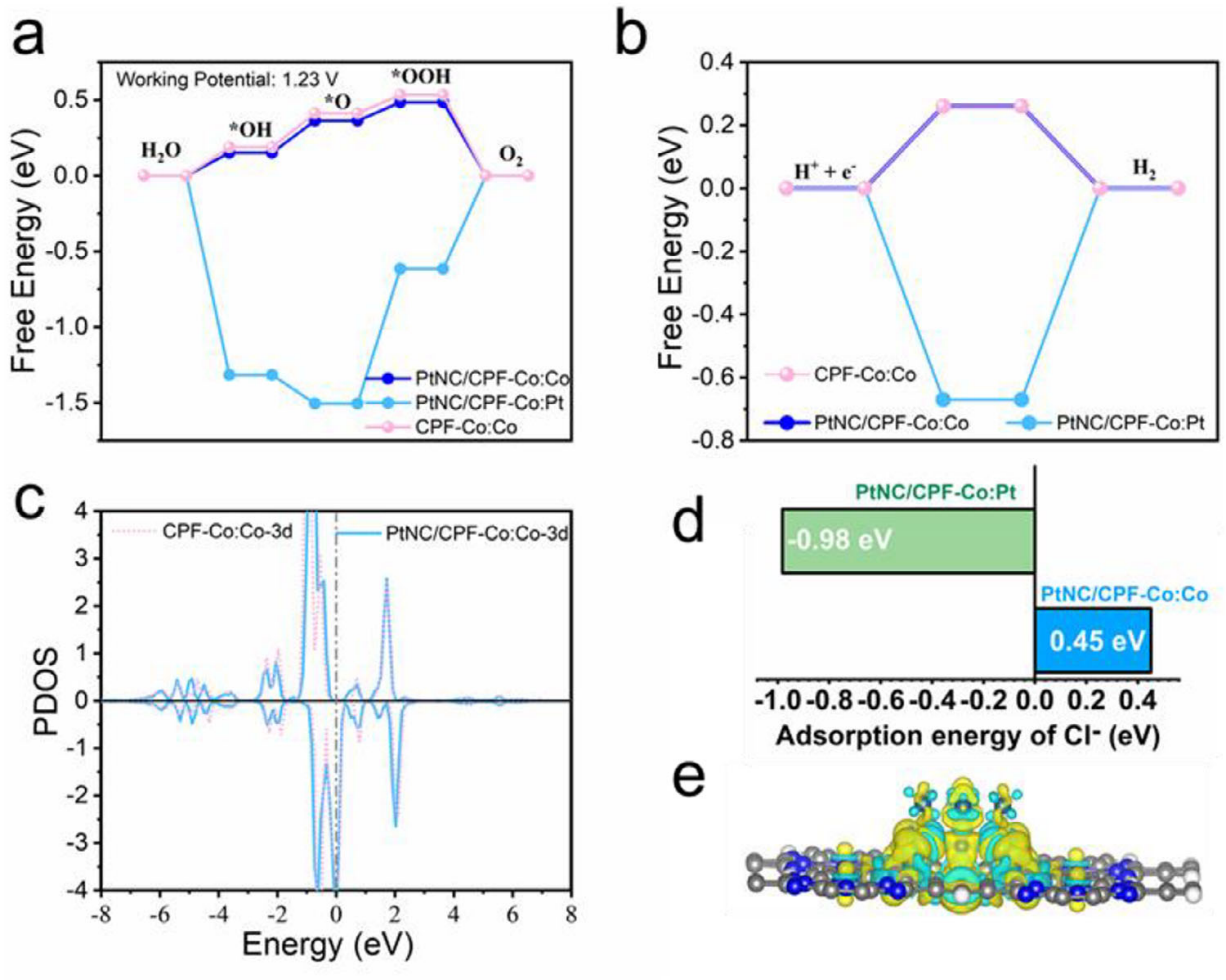
a) Gibbs free energy diagram for the OER reaction of Co sites on CPF‐Co, Co sites on PtNC/CPF‐Co, and Pt sites on PtNC/CPF‐Co. b) Gibbs free energy diagram for the HER reaction of Co sites on CPF‐Co, Co sites on PtNC/CPF‐Co, and Pt sites on PtNC/CPF‐Co. c)The density of state of Co 3d‐orbitals in PtNC/CPF‐Co and CPF‐Co. d) The calculated adsorption energy of Cl^−^ for Pt sites and Co sites on PtNC/CPF‐Co. e) The calculated charge densities of PtNC/CPF‐Co.

From the above phenomenon, it could be concluded that PtNC/CPF‐Co possessed enhanced catalytic ability for both OER and HER compared with the individual Pt nanoclusters or single cobalt sites. Moreover, the cooperative coupling of active sites in the catalysts also demonstrated remarkable selectivity during OER, leading to considerable adaptability in harsh environments. To reveal the synergistic effect and decode the performance improvement of the cooperative coupling Pt‐Co sites, a thorough theoretical calculation was subsequently employed. Based on the Vienna ab‐initio simulation package (VASP), we performed the spin‐polarized density functional theory (DFT) calculations for the activity of OER and HER on PtNC/CPF‐Co and CPF‐Co. Generally, both Pt and Co atoms were able to act as the active center for the reaction. Hence, the free energy change trends of OER/HER were involved and investigated on both Pt and Co sites of PtNC/CPF‐Co (**Figure** **6**; Figures S19 and S20, Supporting Information). According to the analysis, it could be found that on PtNC/CPF‐Co the Co possessed higher activity for OER than that of Pt atom. In comparison, the lower activity of Pt sites was limited by the strong adsorption of the intermediates, which made the free energy become more uphill in *O → *OOH and *OOH → O_2_ processes. These findings indicated that enhanced activity for OER could be attributed to the introduction of Co sites. On the other hand, the Co sites cooperated with PtNC in PtNC/CPF‐Co demonstrated much higher activity in the OER process than that of individual Co in CPF‐Co, which was contributed by the stronger adsorption of intermediates (Figure 6a). Thus, it could be concluded that the embedded Pt clusters could cooperate with the Co sites and regulate the interaction between the intermediates and Co active sites to improve the activity of OER. In addition, for the HER process as shown in Figure 6b, it could be observed that the individual Co sites and cooperative coupling Co sites displayed similar activity in pure CPF‐Co and PtNC/CPF‐Co, respectively. These results further suggested that the PtNC could improve the activity of Co sites for OER, while it resulted in less influence on the activity for HER on Co sites. Notably, during the analysis of PtNC/CPF‐Co, it was found that Pt clusters demonstrated much stronger adsorption of Cl^−^ (free adsorption energy of ‐0.98 eV) than that of the Co site (0.45 eV) (Figure 6d; Figure S21, Supporting Information). These findings suggested that the Pt cluster was able to attract the Cl^−^ during catalysis, thus preventing the Co sites from being poisoned and ensuring the efficient selectivity. In order to gain deeper insights, the electronic structures of CPF‐Co and PtNC/CPF‐Co were employed to depict the physical mechanism of the promoted activity generated by cooperative coupling Pt‐Co sites (Figure 6c). According to the PDOS analysis, the introduced Pt13 would make the PDOS in the spin‐up channel of Co‐3d orbital slightly move to the Fermi level, which could lead to a higher d‐band‐center of Co (‐0.72 eV in PtNC/CPF‐Co vs ‐0.77 eV in pure CPF‐Co). Thus, the adsorption of intermediate species would become stronger and the activity of OER was then improved. The synergistic and moderating effect in the electronic structure could be further proved by the differential charge (Figure 6e; Figure S22, Supporting Information). It could be observed that the introduced PtNC would lose 0.76 e to the CPF‐Co. Such charge redistribution could indirectly affect the electronic structure of Co sites, which acted as the main function and generated the change of the d‐band‐center of Co atom. Hence, our design to assemble the cooperative coupling PtNC‐CoSA active sites simultaneously improved the dual‐catalytic function while enhancing the selectivity, not only preventing the adsorption of Cl^−^ but also leading to considerable adaptability toward saline electrolysis.

## 3 Discussion

In summary, the mild pyrolysis‐free synthetic strategy allowed the directed assembly of cooperative coupling Pt‐Co catalytic sites with precisely controlled electronic configurations. With this advantage, it was able to modulate the intermediate adsorption and charge distributions, thus activating the dual‐catalytic function on the catalysts. The as‐designed PtNC/CPF‐Co demonstrated low overpotentials for both the HER and OER processes in alkaline media. Meanwhile, the synergistic effects of the cooperative coupling active sites also promoted the selectivity of the OER, enabling efficient water‐splitting kinetics and considerable environmental adaptability. PtNC/CPF‐Co exhibited well‐maintained catalytic performance in not only saline water, but also fluctuant and extreme concentrations of hydroxide. From the industrial perspective, pyrolysis‐free synthesis could enable the scale‐up process while eliminating normal energy‐intensive pyrolysis steps. The symmetric PtNC/CPF‐Co||PtNC/CPF‐Co cells further assessed the practical feasibility. More importantly, our synthetic strategy to rational design and precisely control the cooperative coupling catalytic sites could provide rich opportunities for theoretical insights and machine learning‐driven catalysts discovery, bridging the advanced materials innovation and economically viable hydrogen production toward from abundant seawater resources.

## Conflict of Interest

The authors declare no conflict of interest.

## Supporting information



Supporting Information

## Data Availability

The data that support the findings of this study are available in the supplementary material of this article.

## References

[1] J. A. Turner , Science 2004, 305, 972. 15310892 10.1126/science.1103197

[2] S. Chu , A. Majumdar , Nature 2012, 488, 294. 22895334 10.1038/nature11475

[3] H. B. Gray , Nat. Chem. 2009, 1, 7.21378780

[4] Y. Huang , Y. Wang , S. Chen , J. Gao , Y. Wang , Y. Zhang , P. Deng , Aggregate 2023, 4, 335.

[5] K. Yue , R. Lu , M. Gao , F. Song , Y. Dai , C. Xia , B. Mei , H. Dong , R. Qi , D. Zhang , J. Zhang , Z. Wang , F. Huang , B. Y. Xia , Y. Yan , Science 2025, 388, 430. 40273253 10.1126/science.ads1466

[6] Z. H. Li , G. X. Lin , L. Q. Wang , H. Lee , J. Du , T. Tang , G. H. Ding , R. Ren , W. L. Li , X. Cao , S. W. Ding , W. T. Ye , W. X. Yang , L. C. Sun , Nat. Catal. 2024, 7, 944.

[7] J. H. Zhang , X. B. Fu , S. Kwon , K. F. Chen , X. Z. Liu , J. Yang , H. R. Sun , Y. C. Wang , T. Uchiyama , Y. Uchimoto , S. F. Li , Y. Li , X. L. Fan , G. Chen , F. J. Xia , J. S. Wu , Y. B. Li , Q. Yue , L. Qiao , D. Su , H. Zhou , W. A. Goddard , Y. J. Kang , Science 2025, 387, 48. 39745949 10.1126/science.ado9938

[8] H. M. Liu , X. Kang , T. F. Zhao , Z. Y. Zhang , S. Y. Ge , S. Q. Hu , Y. T. Luo , F. N. Yang , S. H. Li , C. H. Sun , Q. M. Yu , H. M. Cheng , B. L. Liu , Sci. China Mater. 2022, 65, 3243.

[9] Y. Kuang , M. J. Kenney , Y. T. Meng , W. H. Hung , Y. J. Liu , J. E. Huang , R. Prasanna , P. S. Li , Y. P. Li , L. Wang , M. C. Lin , M. D. McGehee , X. M. Sun , H. J. Dai , Proc. Natl. Acad. Sci. USA 2019, 116, 6624. 30886092 10.1073/pnas.1900556116 PMC6452679

[10] H. P. Xie , Z. Y. Zhao , T. Liu , Y. F. Wu , C. Lan , W. C. A. Jiang , L. Y. Zhu , Y. P. Wang , D. S. Yang , Z. P. Shao , Nature 2022, 612, 673. 36450987 10.1038/s41586-022-05379-5

[11] G. A. Lindquist , Q. C. Xu , S. Z. Oener , S. W. Boettcher , Joule 2020, 4, 2549.

[12] J. W. Chang , W. Jing , X. Yong , A. Cao , J. K. Yu , H. Wu , C. Z. Wan , S. Y. Wang , G. I. N. Waterhouse , B. Yang , Z. Y. Tang , X. F. Duan , S. Y. Lu , Nat. Synth. 2024, 3, 1427.

[13] W. Liu , J. Yu , M. G. Sendeku , T. S. Li , W. Q. Gao , G. T. Yang , Y. Kuang , X. M. Sun , Angew. Chem., Int. Ed. 2023, 62, 7. 10.1002/anie.202309882 37603411

[14] Y. Gao , Y. R. Xue , L. Qi , C. Y. Xing , X. C. Zheng , F. He , Y. L. Li , Nat. Commun. 2022, 13, 11. 36064713 10.1038/s41467-022-32937-2 PMC9445080

[15] S. Dresp , F. Dionigi , M. Klingenhof , P. Strasser , ACS Energy Lett. 2019, 4, 933.

[16] W. M. Tong , M. Forster , F. Dionigi , S. Dresp , R. S. Erami , P. Strasser , A. J. Cowan , P. Farràs , Nat. Energy 2020, 5, 367.

[17] R. Ram , L. Xia , H. Benzidi , A. Guha , V. Golovanova , A. G. Manjón , D. L. Rauret , P. S. Berman , M. Dimitropoulos , B. Mundet , E. Pastor , V. Celorrio , C. A. Mesa , A. M. Das , A. Pinilla‐Sánchez , S. Giménez , J. Arbiol , N. López , F. P. G. de Arquer , Science 2024, 384, 1373. 38900890 10.1126/science.adk9849

[18] Z. Xu , Z.‐S. Wu , eScience 2024, 5, 100334.

[19] G. Janani , S. Surendran , D.‐K. Lee , S. Shanmugapriya , H. Lee , Y. Subramanian , U. Sim , Aggregate 2024, 5, 430.

[20] T. P. Keane , S. S. Veroneau , A. C. Hartnett , D. G. Nocera , J. Am. Chem. Soc. 2023, 145, 4989. 36848225 10.1021/jacs.3c00176

[21] W. Shi , T. Shen , C. Xing , K. Sun , Q. Yan , W. Niu , X. Yang , J. Li , C. Wei , R. Wang , S. Fu , Y. Yang , L. Xue , J. Chen , S. Cui , X. Hu , K. Xie , X. Xu , S. Duan , Y. Xu , B. Zhang , Science 2025, 387, 791. 39946454 10.1126/science.adr3149

[22] F. S. Hegner , F. A. Garcés‐Pineda , J. González‐Cobos , B. Rodríguez‐García , M. Torréns , E. Palomares , N. López , J. R. Galán‐Mascarós , ACS Catal. 2021, 11, 13140.

[23] J. Zhao , Y. Guo , Z. Q. Zhang , X. L. Zhang , Q. Q. Ji , H. Zhang , Z. Q. Song , D. Q. Liu , J. R. Zeng , C. H. Chuang , E. R. Zhang , Y. H. Wang , G. Z. Hu , M. A. Mushtaq , W. Raza , X. K. Cai , F. Ciucci , Nat. Nanotechnol. 2025, 20, 12. 39433919 10.1038/s41565-024-01807-x PMC11750697

[24] Y. L. Xing , J. G. Ku , W. Fu , L. Z. Wang , H. H. Chen , Chem. Eng. J. 2020, 395, 7.

[25] M. Li , Y. Deng , G. Wu , S. Xue , Y. Yan , Z. Liu , J. Zou , D. Yang , A. Dong , Aggregate 2021, 2, 17.

[26] H. Liu , R. Xie , Y. Luo , Z. Cui , Q. Yu , Z. Gao , Z. Zhang , F. Yang , X. Kang , S. Ge , S. Li , X. Gao , G. Chai , L. Liu , B. Liu , Nat. Commun. 2022, 13, 6382. 36289229 10.1038/s41467-022-34121-y PMC9605970

[27] F. N. Yang , Y. T. Luo , Q. M. Yu , Z. Y. Zhang , S. Zhang , Z. B. Liu , W. C. Ren , H. M. Cheng , J. Li , B. L. Liu , Adv. Funct. Mater. 2021, 31, 10.

[28] aX. Kang , F. N. Yang , Z. Y. Zhang , H. M. Liu , S. Y. Ge , S. Q. Hu , S. H. Li , Y. T. Luo , Q. M. Yu , Z. B. Liu , Q. Wang , W. C. Ren , C. H. Sun , H. M. Cheng , B. L. Liu , Nat. Commun. 2023, 14, 10.36599855

[29] W. W. Xu , Z. F. Wang , P. Y. Liu , X. Tang , S. X. Zhang , H. C. Chen , Q. H. Yang , X. Chen , Z. Q. Tian , S. Dai , L. Chen , Z. Y. Lu , Adv. Mater. 2024, 36, 8. 10.1002/adma.202306062 37907201

[30] C. Q. Huang , Q. C. Zhou , L. Yu , D. S. Duan , T. Y. Cao , S. H. Qiu , Z. Z. Wang , J. Guo , Y. X. Xie , L. P. Li , Y. Yu , Adv. Energy Mater. 2023, 13, 12.

[31] L. Yu , Q. Zhu , S. W. Song , B. McElhenny , D. Z. Wang , C. Z. Wu , Z. J. Qin , J. M. Bao , Y. Yu , S. Chen , Z. F. Ren , Nat. Commun. 2019, 10, 10. 31704926 10.1038/s41467-019-13092-7 PMC6841982

[32] B. Zhang , X. L. Zheng , O. Voznyy , R. Comin , M. Bajdich , M. García‐Melchor , L. L. Han , J. X. Xu , M. Liu , L. R. Zheng , F. P. G. de Arquer , C. T. Dinh , F. J. Fan , M. J. Yuan , E. Yassitepe , N. Chen , T. Regier , P. F. Liu , Y. H. Li , P. De Luna , A. Janmohamed , H. L. L. Xin , H. G. Yang , A. Vojvodic , E. H. Sargent , Science 2016, 352, 333. 27013427 10.1126/science.aaf1525

[33] X. R. Zhang , C. Y. Sun , S. S. Xu , M. R. Huang , Y. Wen , X. R. Shi , Nano Res. 2022, 15, 8897.

[34] C. J. Huang , H. M. Xu , T. Y. Shuai , Q. N. Zhan , Z. J. Zhang , G. R. Li , Appl. Catal., B 2023, 325, 33.

[35] M. Batool , A. Hameed , M. A. Nadeem , Coord. Chem. Rev. 2023, 480, 29.

[36] G. H. Gao , R. Z. Zhao , Y. J. Wang , X. Ma , Y. Li , J. Zhang , J. S. Li , Chin. Chem. Lett. 2024, 35, 5.

[37] J. Ni , Z. P. Shi , Y. B. Wang , J. H. Yang , H. X. Wu , P. B. Wang , M. L. Xiao , C. P. Liu , W. Xing , eScience 2025, 5, 19.

[38] J. X. Guo , Y. Zheng , Z. P. Hu , C. Y. Zheng , J. Mao , K. Du , M. Jaroniec , S. Z. Qiao , T. Ling , Nat. Energy 2023, 8, 264.

[39] L. L. Wang , J. Y. Diao , M. Peng , Y. L. Chen , X. B. Cai , Y. C. Deng , F. Huang , X. T. Qin , D. Q. Xiao , Z. Jiang , N. Wang , T. Sun , X. D. Wen , H. Y. Liu , D. Ma , ACS Catal. 2021, 11, 11469.

[40] W. Z. Huang , J. T. Li , X. B. Liao , R. H. Lu , C. H. Ling , X. Liu , J. S. Meng , L. B. Qu , M. T. Lin , X. F. Hong , X. B. Zhou , S. L. Liu , Y. Zhao , L. Zhou , L. Q. Mai , Adv. Mater. 2022, 34, 8.

[41] Z. Li , Y. E. Zhou , M. H. Xie , H. Cheng , T. Wang , J. Chen , Y. Lu , Z. L. Tian , Y. Q. Lai , G. H. Yu , Angew. Chem., Int. Ed. 2023, 62, 9. 10.1002/anie.202217815 36988032

[42] Y. K. Lv , Y. Han , K. Wang , W. Y. Sun , C. X. Du , R. W. Huang , P. Peng , S. Q. Zang , ACS Nano 2024, 18, 32186. 39495627 10.1021/acsnano.4c11554

[43] Y. D. Hu , G. Luo , L. G. Wang , X. K. Liu , Y. T. Qu , Y. S. Zhou , F. Y. Zhou , Z. J. Li , Y. F. Li , T. Yao , C. Xiong , B. Yang , Z. Q. Yu , Y. Wu , Adv. Energy Mater. 2021, 11, 8.

[44] B. Tang , X. L. Zhang , Q. Q. Ji , M. Ge , H. J. Wang , R. Q. Liu , P. Jiang , H. J. Liu , C. Wang , H. Tan , W. S. Yan , ACS Catal. 2024, 14, 3788.

[45] L. A. Chong , G. P. Gao , J. G. Wen , H. X. Li , H. P. Xu , Z. Green , J. D. Sugar , A. J. Kropf , W. Q. Xu , X. M. Lin , H. Xu , L. W. Wang , D. J. Liu , Science 2023, 380, 609. 37167381 10.1126/science.ade1499

[46] Y. Zang , D. Q. Lu , K. Wang , B. Li , P. Peng , Y. Q. Lan , S. Q. Zang , Nat. Commun. 2023, 14, 9. 36997545 10.1038/s41467-023-37530-9 PMC10063682

[47] X. Q. Mu , X. Y. Gu , S. P. Dai , J. B. Chen , Y. J. Cui , Q. Chen , M. Yu , C. Y. Chen , S. L. Liu , S. C. Mu , Energy Environ. Sci. 2022, 15, 4048.

[48] Z. D. Wang , Y. Han , Y. Y. Wang , S. Q. Zang , P. Peng , Angew. Chem., Int. Ed. 2025, 64, 7. 10.1002/anie.202416973 39503347

[49] A. De Backer , G. T. Martinez , A. Rosenauer , S. Van Aert , Ultramicroscopy 2013, 134, 23. 23759467 10.1016/j.ultramic.2013.05.003

[50] L. N. Cao , W. Liu , Q. Q. Luo , R. T. Yin , B. Wang , J. Weissenrieder , M. Soldemo , H. Yan , Y. Lin , Z. H. Sun , C. Ma , W. H. Zhang , S. Chen , H. W. Wang , Q. Q. Guan , T. Yao , S. Q. Wei , J. L. Yang , J. L. Lu , Nature 2019, 565, 631. 30700869 10.1038/s41586-018-0869-5

[51] G. H. Major , V. Fernandez , N. Fairley , E. F. Smith , M. R. Linford , J. Vac. Sci. Technol. A 2022, 40, 10.

[52] R. Tholkappiyan , K. Vishista , Appl. Surf. Sci. 2015, 351, 1016.

[53] M. C. Biesinger , B. P. Payne , A. P. Grosvenor , L. W. M. Lau , A. R. Gerson , R. S. Smart , Appl. Surf. Sci. 2011, 257, 2717.

[54] Y. M. Zhu , J. A. Wang , T. Koketsu , M. Kroschel , J. M. Chen , S. Y. Hsu , G. Henkelman , Z. W. Hu , P. Strasser , J. W. Ma , Nat. Commun. 2022, 13, 12. 36517475 10.1038/s41467-022-35426-8 PMC9751110

[55] J. Bauer , D. H. Buss , H. J. Harms , O. Glemser , J. Electrochem. Soc. 1990, 137, 173.

[56] Y. Liu , S. S. Zhang , C. Jiao , H. M. Chen , G. Wang , W. J. Wu , Z. W. Zhuo , J. J. Mao , Adv. Sci. 2023, 10, 7. 10.1002/advs.202206107 PMC9929106 36494096

[57] A. C. Lazanas , M. I. Prodromidis , ACS Meas , Sci. Au 2023, 3, 162. 10.1021/acsmeasuresciau.2c00070 PMC10288619 37360038

