# Supplementary material for: Pyrolysis‐Free Formed Cooperative Coupling Pt‐Co Dual‐Functional Sites for Saline Electrolysis with High Adaptability
Source: Adv Sci (Weinh). 2025 Oct 7;12(48):e09394. doi: 10.1002/advs.202509394 (PMC12752562; doi:10.1002/advs.202509394)
Supplement: Supplementary file 1 — Supporting Information [file ADVS-12-e09394-s001.pdf]

Supporting information

Pyrolysis-free formed cooperative Pt-Co dual-functional sites for saline electrolysis with high adaptability

Section A. Supporting method.

Chemicals and materials. All chemicals and reagents used in this research were purchased commercially and used without further purification. Water used in this work was purified using the Milli-Q purification system. $H_2PtCl_6·6H_2O$, $Co(NO_3)_2·H_2O$, KCl, Nafion solution, potassium hydroxide (99.99% trace metals basis, KOH) were purchased from Sigma-Aldrich. Commercial Pt/C catalyst (20 wt% loading) and RuO_2 was purchased from Johnson Matthey. Deionized (DI) water with a sensitivity of 18.2 MΩ cm^{-1} from the ELGA CHORUS 1 (Chemoscience Pte Ltd) was used all the time in this work. Hydrophilic carbon papers were treated before use, using 10% nitric acid and 10% sulfuric acid mixed solution immersion treatment, and then rinse with deionized water three times. All the chemical reagents used in the experiment were analytically pure.

Synthesis of CPF-Co. 1,2,4,5-tetracyanobenzene, $Co(NO_3)_2·H_2O$ and 1,8-Diazabicyclo (5,4,0) undec-7-ene (DBU) were dissolved in N,N-Dimethylformamide (10 mL) in a 20 mL screw glass vial and heated at 180 °C for 4 days. After cooled to room temperature, a dark solid of CPF-Co were formed. After filtration and washed by DMF, the wet sample was transferred to a Soxhlet extractor and washed with THF (24 h) and acetone (24 h). The obtained solid was dried in the vacuum oven at 60 °C for 12 h and then characterized thoroughly. The sample was also used directly for the photocatalysis without any further purification.

Synthesis of PtNC/CPF-Co. 100 mg CPF-Co and 5 g KOH were dissolved in a mixture of 100 mL DMF/ ethylene glycol (v:v=1:9) and 1 mL H2PtCl6 was dissolved in 50 mL DMF/ ethylene glycol (v:v=1:9) in the mixture of solvent, 10

mL of the above mixed solution was taken into the CPF-Co reaction solution, and the microwave reaction was conducted at 400 W power for 1 h, 180℃; the remaining 40 mL of the mixed solvent was placed in the CPF-Co reaction solution, and the microwave reaction was continued at 400 W power for 1 h, 180℃.

Characterization techniques. The diffractograms of PXRD were measured with Cu Kα1 radiation (λ= 1.540598 Å) on a PANalytical EMPYREAN diffractometer at room temperature. FT-IR spectras were performed with KBr pellets in the range 400-4000 cm^{-1} on a Nexus 870 FTIR spectrometer.X-ray photoelectron spectroscopy (XPS) were performed on a Thermo ESCALAB 250XI electron spectrometer using 300 W Al K radiation. Inductively coupled plasma mass spectra (ICP-MS) were recorded with Shimadzu ICP-OES Agilent 5110. The X-ray absorption find structure spectra (Cu K-edge) were collected at 1W1B beamline of Beijing Synchrotron Radiation Facility (BSRF). The data were collected in transmission mode using a Lytle detector. The samples were grinded and uniformly daubed on the special adhesive tape. The energy range of 4B9A and 1w2A beam line is 5-20 keV. The energy range of monochromator is better than $3*10^3$ in this line. The intensity range of Beam line is $2×10^9$-$2×10^{10}$cps. The spot size at the sample is 0.8*0.9 mm^2. The morphology of the samples was observed by transmission electron microscopy (TEM, FEI TalosF 200S). HAADF-STEM images and energy-dispersive X-ray Spectroscopy (EDS) elemental mapping were conducted on a JEOL JEM-ARM300F STEM/TEM with a guaranteed resolution of 0.08 nm.

Electrochemical measurements. The electrochemical measurements were accomplished at MULTI AUTOLAB M204 electrochemical workstation. Carbon paper, Platinum tablets and graphite rod were used as working electrode, counter electrode and reference electrodes. All potentials were calibrated relatively to reversible hydrogen electrode (RHE) with iR compensation. For preparation of the working electrode, 3 mg catalyst were dispersed into 270 uL DMF and 30 μL Nafion (5%) under sonication for 30 min to form a

homogeneous ink. Then, 10 μL catalyst ink was dropped onto the carbon paper and dried naturally. All potentials were converted to the RHE reference scale using the relation $E_{RHE} = E_{Ag/AgCl}$ + pH×0.059 +0.197. To evaluate the HER activities of as-prepared catalysts, the scan rate of linear sweep voltammetry (LSV) was set to 5.0 mV s^{-1}. The electrochemical impedance spectroscopy (EIS) measurement was performed in a frequency range from 0.01 to 100000 Hz by applying an AC voltage with 5 mV amplitude.

Computational Details. The Vienna ab-initio simulation package (VASP) [1-5] is employed to perform the spin-polarized density functional theory (DFT) calculations in this work. The core electrons of atoms were treated by Blöchl's all-electron-like projector augmented wave (PAW) method [5,6]. The Perdew-Burke-Ernzerbof (PBE) within the generalized gradient approximation functional (GGA) is adopted to describe the exchange and correlation effects [7]. The plane wave energy cutoff was set as 400 eV. The Gaussian scheme was employed for electron occupancy with an energy smearing of 0.1 eV. The first Brillouin zone was sampled in the Monkhorst−Pack grid [8]. The 3×3×1 k-point mesh is used for the calculations. The energy (converged to 1.0 ×10^{-6} eV/atom) and force (converged to 0.01eV/Å) were set as the convergence criterion for geometry optimization. To meet the experimental configuration, an 2×2 CPF-Co is built with a Pt_{13} cluster anchored in the hollow site. A vacuum layer of 20 Å was employed along the c axis to avoid the periodic interaction. A pure CPF-Co with 2×2 supercell was also employed for comparison.

The free energy change of the elementary reaction in OER is estimated by the following expression [9]:

$$\Delta G = \Delta E + \Delta ZEP - T\Delta S + \Delta G_U + \Delta G_{pH} + \Delta G_{field} \qquad (1)$$

where *ΔE* is the total energy change from initial states to final states of the reaction obtained by DFT calculations. *ΔZPE* is the change in zero-point energy. *T* is room temperature (298.15 K). *ΔS* is the change in entropy. *ΔG=-eU*, where *U* is the electrode potential with respect to standard hydrogen

electrode, and *e* is the transferred charge. $\Delta G_{pH} = k_B T \ln 10 \times pH$ where k_B is the Boltzmann constant, and pH=0 for acid medium [10,11]. ΔG_{field} is the free energy correction due to the electrochemical double layer and is neglected as in previous studies [9,11]. Gas-phase H_2O at 0.035 bar was used as the reference state, which is the equilibrium point of the gas-phase and liquid water at 298.15 K. The free energy of O_2 is obtained from the free energy change of the reaction $2H_2O \rightarrow O_2 + 2H_2$, which is 4.92 eV at 298.15 K and a pressure of 0.035 bar [9]. The free energy of ($H^+ + e^-$) in solution at standard conditions of pH=0 and U=0 is equal to that of $1/2H_2$ according to a computational hydrogen electrode model suggested by Nørskov et al [9]. The entropy of the H_2 is taken from the National Institute of Standards and Technology (NIST) database [12], while the entropies of the OER intermediates were calculated from the vibrational frequencies.

The working potential is the potential that keeps all the elementary reactions to be exothermic. It is defined as:

$$U_{OER}=\max[\Delta G_x]/ne \quad (2)$$

where n is the number of electrons transferred for each reaction, and e is the elementary charge [13,14].

EXAFS analysis. Data reduction, data analysis, and EXAFS fitting is applied through Athena and Artemis software.[15] The energy calibration of the sample was conducted through a standard foil, which as a reference was simultaneously measured. For EXAFS modeling, The global amplitude EXAFS (*CN, R,* σ^2 and ΔE_0) were obtained by nonlinear fitting, with least-squares refinement, of the EXAFS equation to the Fourier-transformed data in R-space, using Artemis software, EXAFS of the foil is fitted and the obtained amplitude reduction factor S_0^2 value was set in the EXAFS analysis to determine the coordination numbers (CNs) in the scattering path in sample. The Debye-Waller factors and delta *R*s are obtained based on the *guessing* parameters and constrained for paths. Wavelet transformation (WT) is also

employed using the software package developed by Funke and Chukalina using Morlet wavelet with κ = 10, σ = 1.[16,17]

References

[1] G. Kresse, J. Furthmüller, Efficiency of Ab-initio total energy calculations for metals and semiconductors using a plane-wave basis set, Comp. Mater. Sci. 6 (1996) 15–50.

[2] G. Kresse, J. Hafner, Ab initio molecular dynamics for liquid metals, Phys. Rev. B 47 (1993) 558–561.

[3] G. Kresse, J. Hafner, Ab initio molecular-dynamics simulation of the liquid-metal–amorphous-semiconductor transition in germanium, Phys. Rev. B 49, (1994) 14251–14269.

[4] G. Kresse, J. Furthmüller, Efficient iterative schemes for ab initio total-energy calculations using a plane-wave basis set, Phys. Rev. B 54 (1996) 11169–11186.

[5] P. E. Blöchl, Projector augmented-wave method, Phys. Rev. B 50 (1994)17953–17979.

[6] G. Kresse, D. Joubert, From ultrasoft pseudopotentials to the projector augmented-wave method, Phys. Rev. B 59 (1999) 1758–1775.

[7] J. P. Perdew, K. Burke, M. Ernzerhof, Generalized gradient approximation made simple, Phys. Rev. Lett. 77 (1996) 3865–3868.

[8] H. J. Monkhorst, J. D. Pack, Special points for brillouin-zone integrations, Phys. Rev. B 13 (1976) 5188–5192.

[9] J. K. Nørskov, J. Rossmeisl, A. Logadottir, L. Lindqvist, J. R. Kitchin, T. Bligaard, H. Jónsson, Origin of the overpotential for oxygen reduction at a fuel-cell cathode, J. Phys. Chem. B 108 (2004) 17886–17892.

[10] L. Yu, X. Pan, X. Cao, P. Hu, X. Bao, Oxygen reduction reaction mechanism on nitrogen-doped graphene: A density functional theory study, J. Catal. 282 (2011) 183-190.

[11] S. Kattel, P. Atanassov, B. Kiefer, Density functional theory study of Ni–N_x/C electrocatalyst for oxygen reduction in alkaline and acidic media, J. Phys. Chem. C 116 (2012) 17378–17383.

[12] J. D. Cox, D. D. Wagman, V. A. Medvedev, CODATA key values for thermodynamics, Hemisphere Publishing Corp., New York, 1984, 1.

[13] G. L. Chai, K. P. Qiu, M. Qiao, M. M. Titirici, C. X. Shang, Z. X. Guo, Active sites engineering leads to exceptional ORR and OER bifunctionality in P,N Co-doped graphene frameworks, Energy Environ. Sci.10 (2017) 1186−1195

[14] G. L. Chai, Z. F. Hou, D. J. Shu, T. Ikeda, K. Terakura, Active sites and mechanisms for oxygen reduction reaction on nitrogen-doped carbon alloy catalysts: Stone−wales defect and curvature effect, J. Am. Chem. Soc. 136 (2014) 13629–13640.

[15] Ravel, B., & Newville, M. (2005). ATHENA, ARTEMIS, HEPHAESTUS: data analysis for X-ray absorption spectroscopy using IFEFFIT. *Journal of synchrotron radiation*, 12(4), 537-541.
[16] Funke, H., Scheinost, A. C., & Chukalina, M. (2005). Wavelet analysis of extended x-ray absorption fine structure data. *Physical Review B*, 71(9), 094110.
[17] Funke, H., Chukalina, M., & Scheinost, A. C. (2007). A new FEFF-based wavelet for EXAFS data analysis. *Journal of synchrotron radiation*, 14(5), 426-432.

Section B. Supporting data.

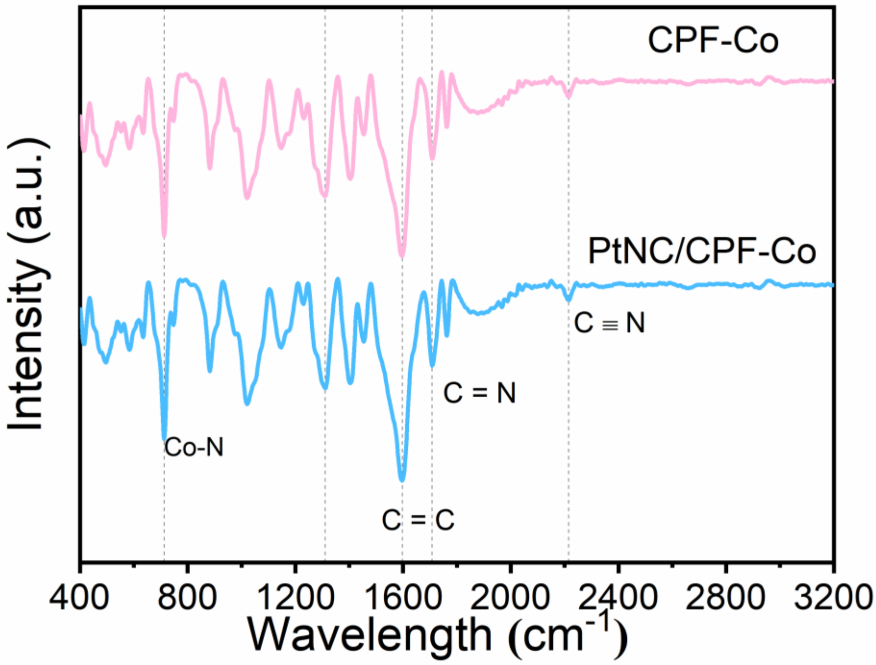

Figure S1. FT-IR spectrums of CPF-Co and PtNC/CPF-Co.

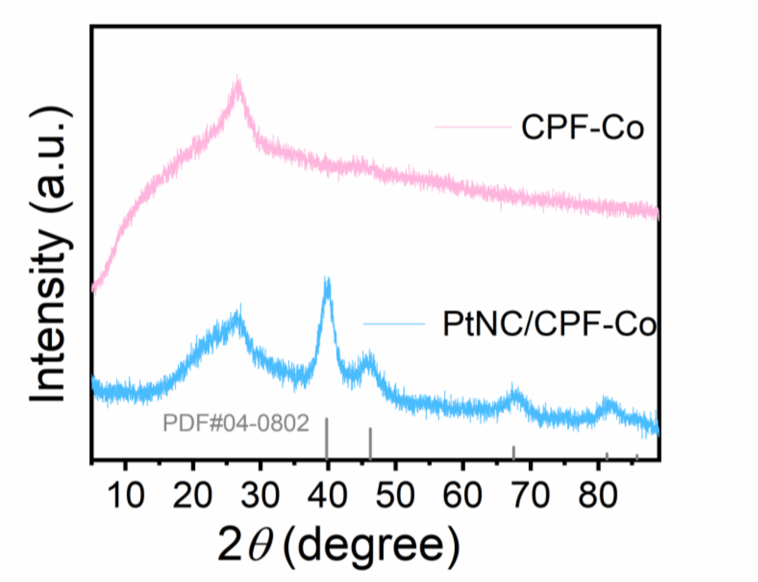

Figure S2. PXRD spectra of CPF-Co and PtNC/CPF-Co.

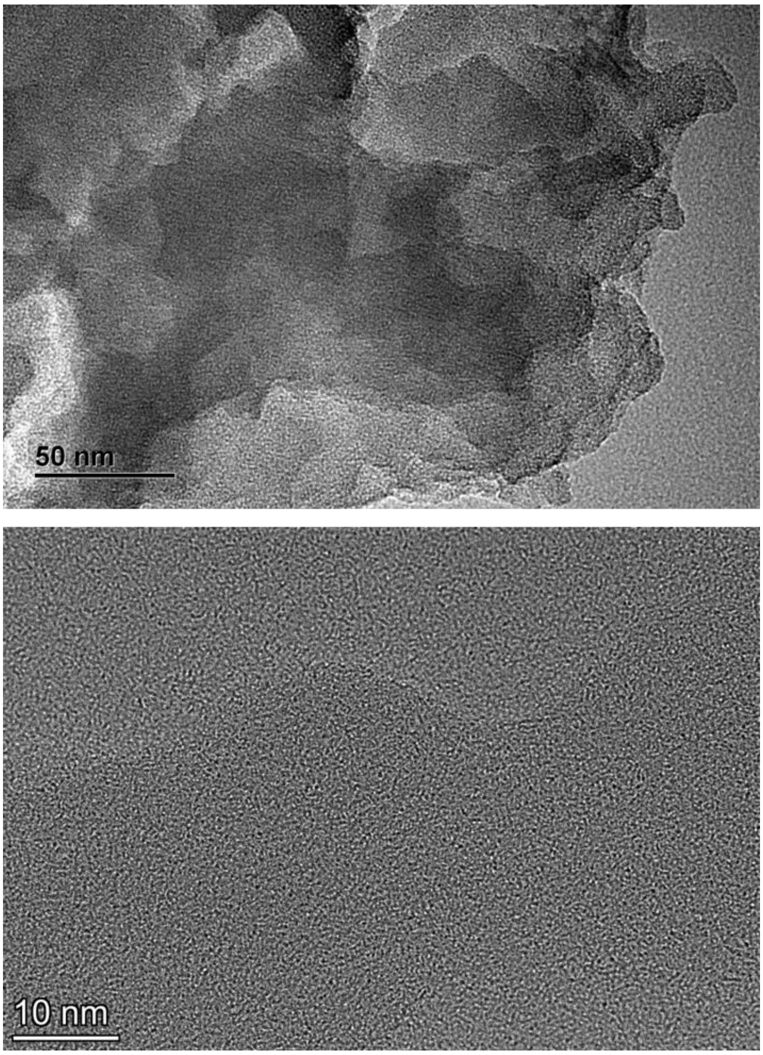

Figure S3 Transmission electron microscopy (TEM) images of CPF-Co.

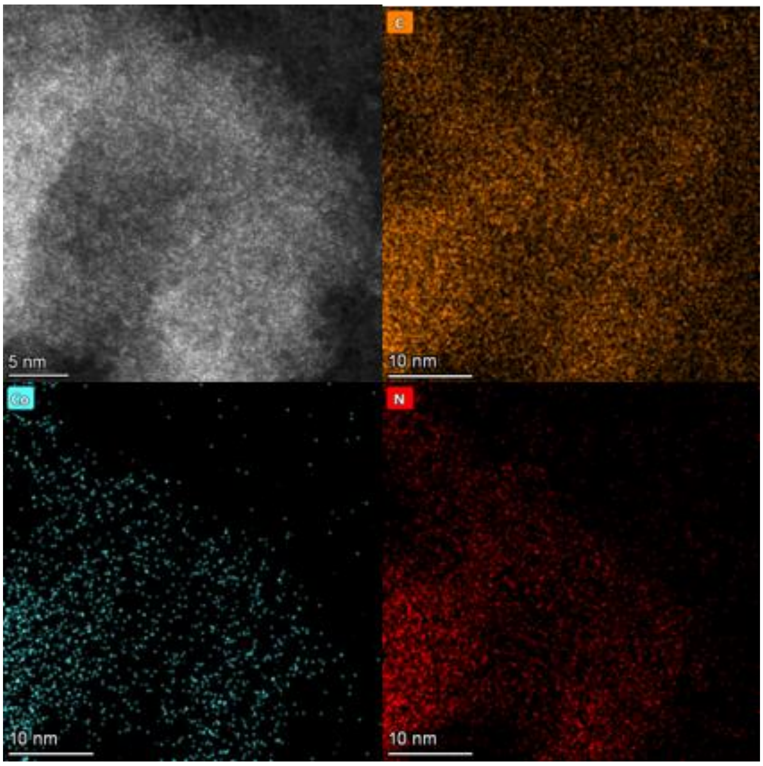

Figure S4 EDS elemental mapping images of CPF-Co.

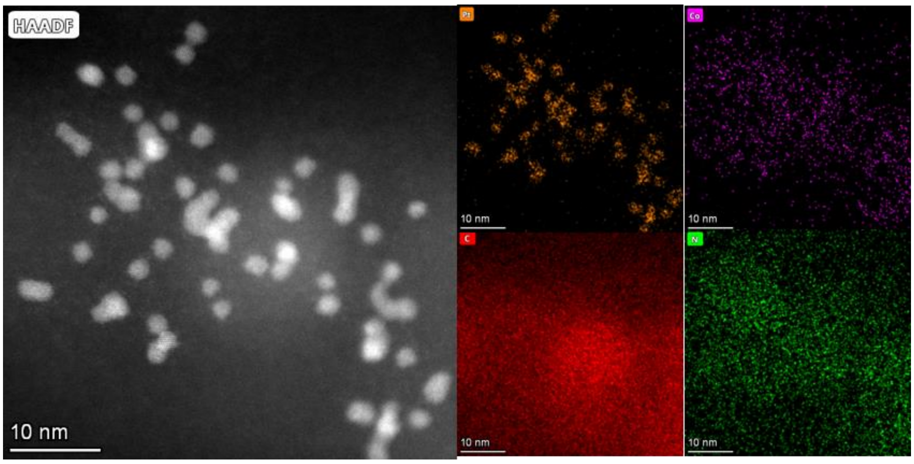

Figure S5 EDS elemental mapping images of PtNC/CPF-Co.

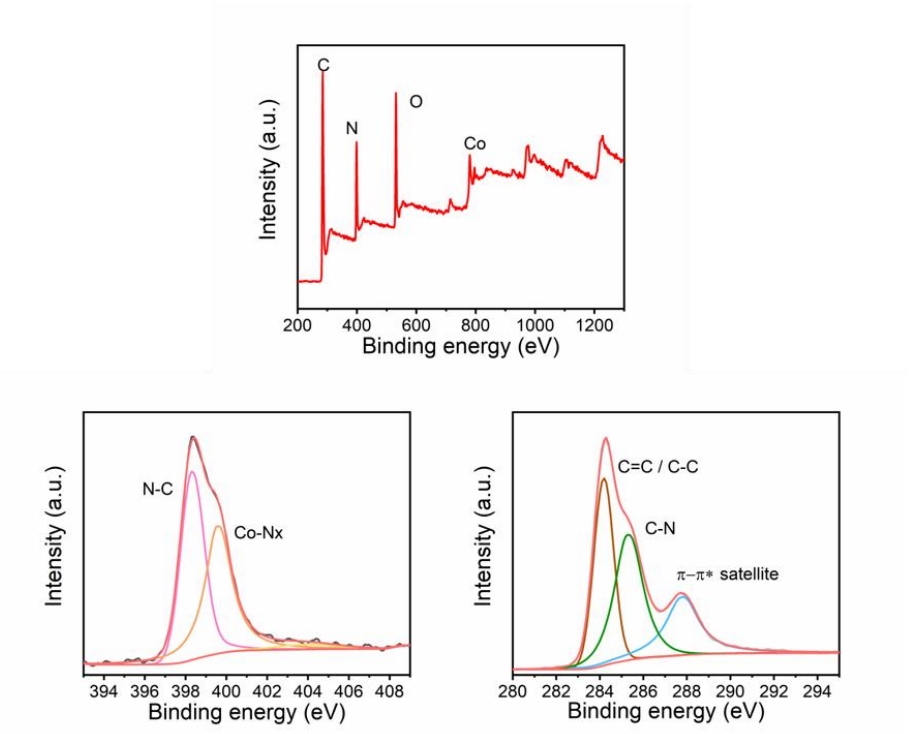

Figure S6 XPS spectra of CPF-Co.

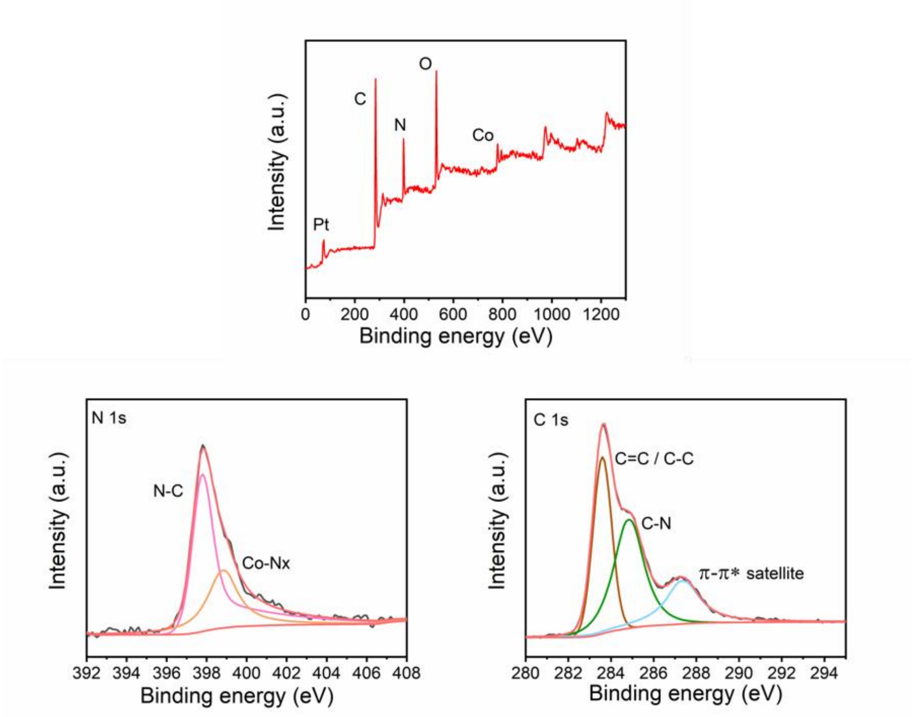

Figure S7 XPS spectra of PtNC/CPF-Co.

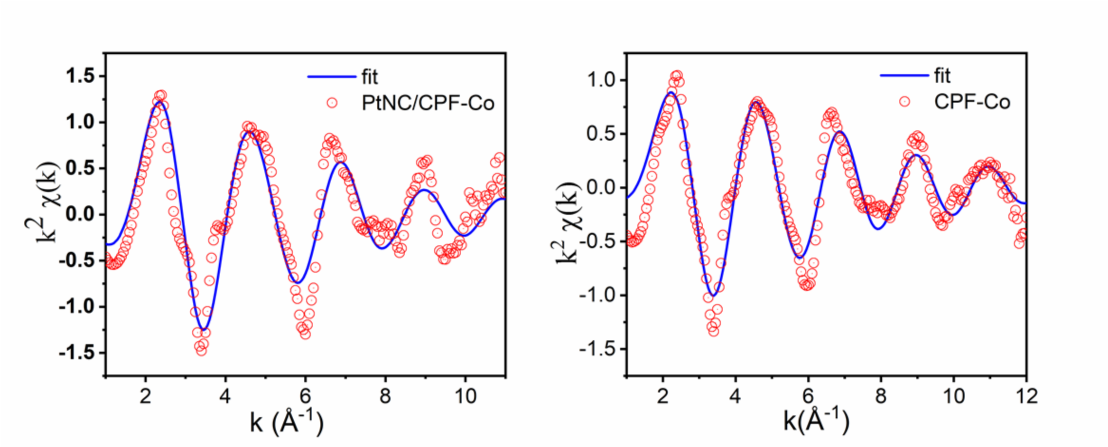

Figure S8 Fourier-transformed magnitude of Co K EXAFS spectra in k space.

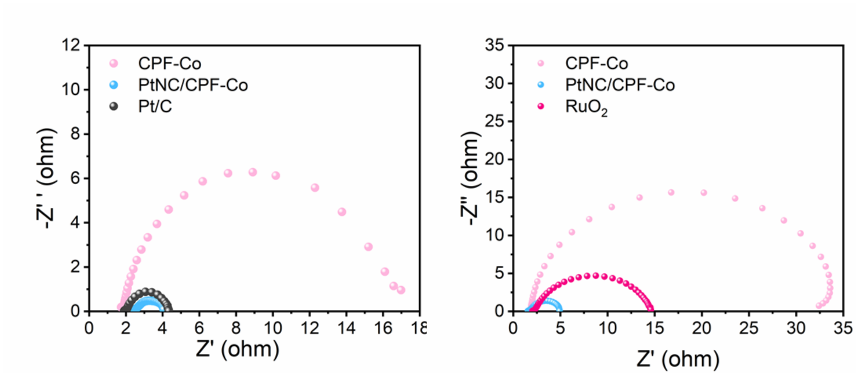

Figure S9 (left) Comparison of CPF-Co, PtNC/CPF-Co and commercial Pt/C for HER; (right) Comparison of CPF-Co, PtNC/CPF-Co and commercial RuO_2 for OER.

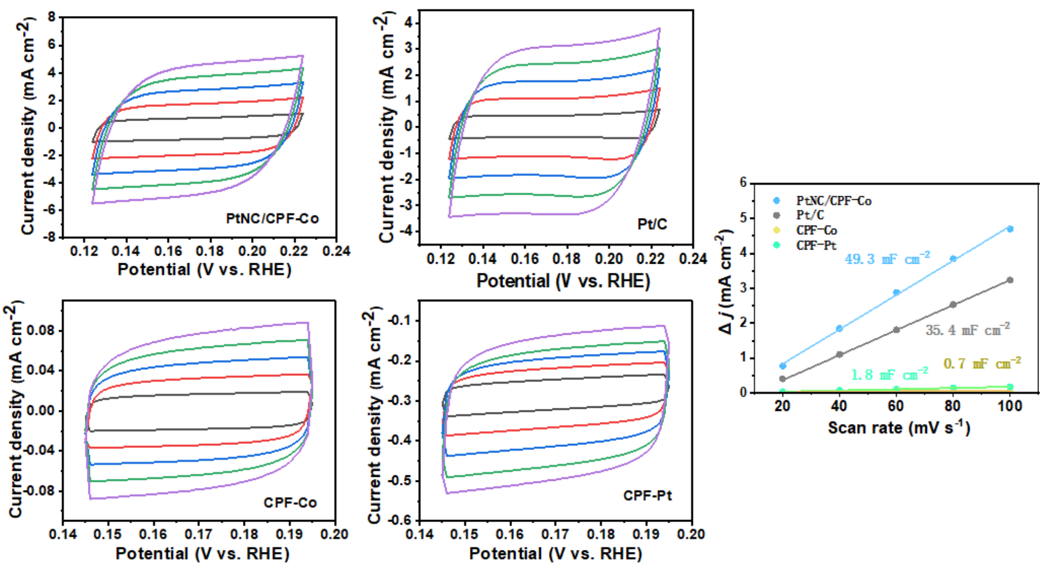

Figure S10 CV curves of as prepared catalysts at scan rates ranging from rates of 20, 40, 60, 80 and 100 mV s^{-1} outside HER region in 1 M KOH; ECSA of prepared catalysts in 1 M KOH.

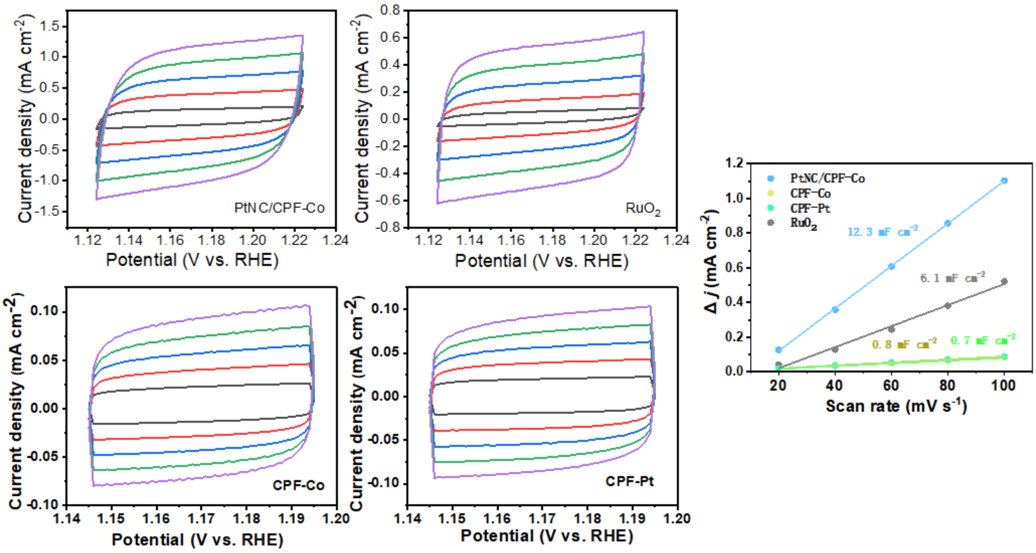

Figure S11 CV curves of as prepared catalysts at scan rates ranging from rates of 20, 40, 60, 80 and 100 mV s^{-1} outside OER region in 1 M KOH; ECSA of prepared catalysts in 1 M KOH.

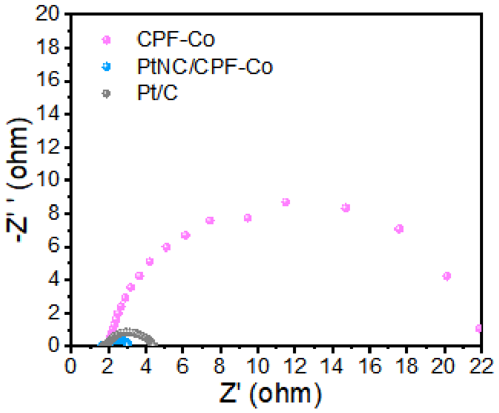

Figure S12 Comparison of CPF-Co, PtNC/CPF-Co and commercial Pt/C in 1 M KOH + 0.5 M NaCl.

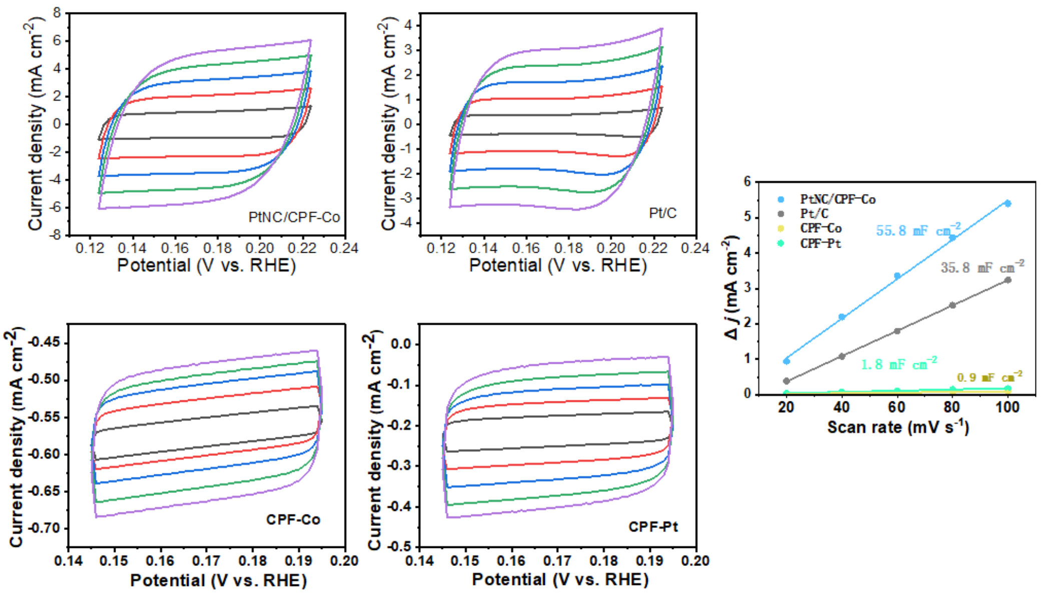

Figure S13 CV curves of as prepared catalysts at scan rates ranging from rates of 20, 40, 60, 80 and 100 mV s^{-1} outside HER region in 1 M KOH + 0.5 M NaCl; ECSA of prepared catalysts in 1 M KOH + 0.5 M NaCl.

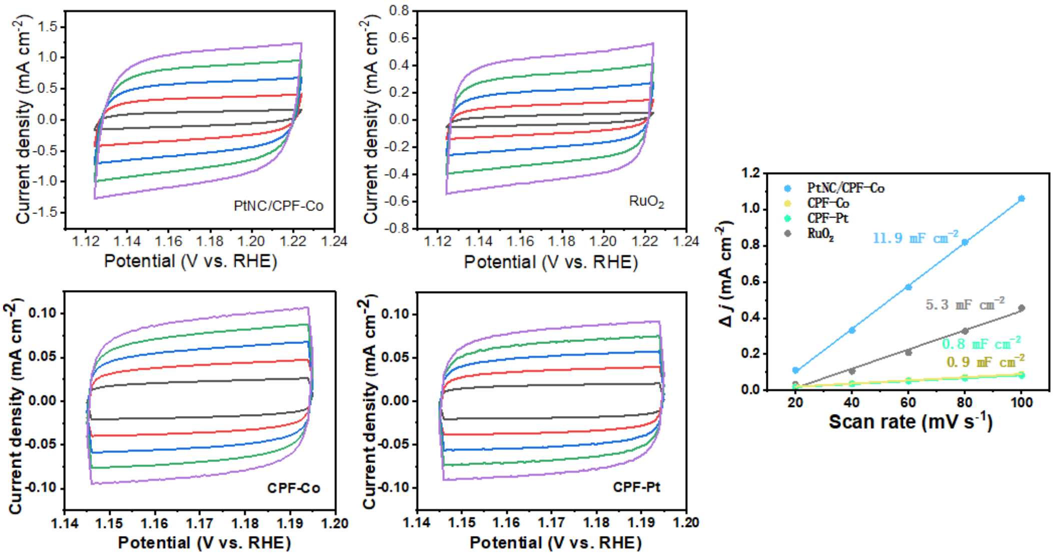

Figure S14 CV curves of as prepared catalysts at scan rates ranging from rates of 20, 40, 60, 80 and 100 mV s^{-1} outside OER region in 1 M KOH + 0.5 M NaCl; ECSA of prepared catalysts in 1 M KOH + 0.5 M NaCl.

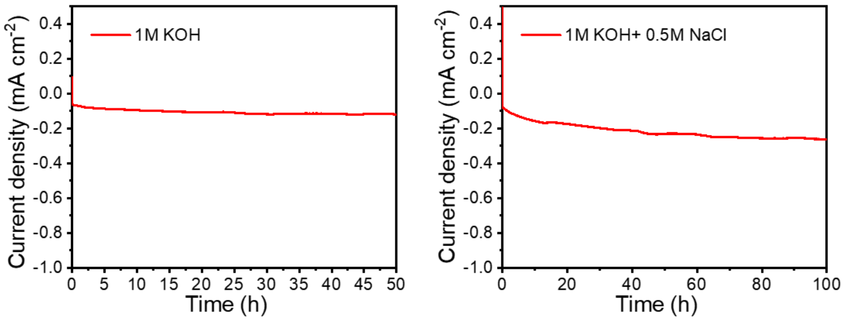

Figure S15 The chronoamperometry tests of PtNC/CPF-Co catalysts in 1 M KOH with and without 0.5 M NaCl.

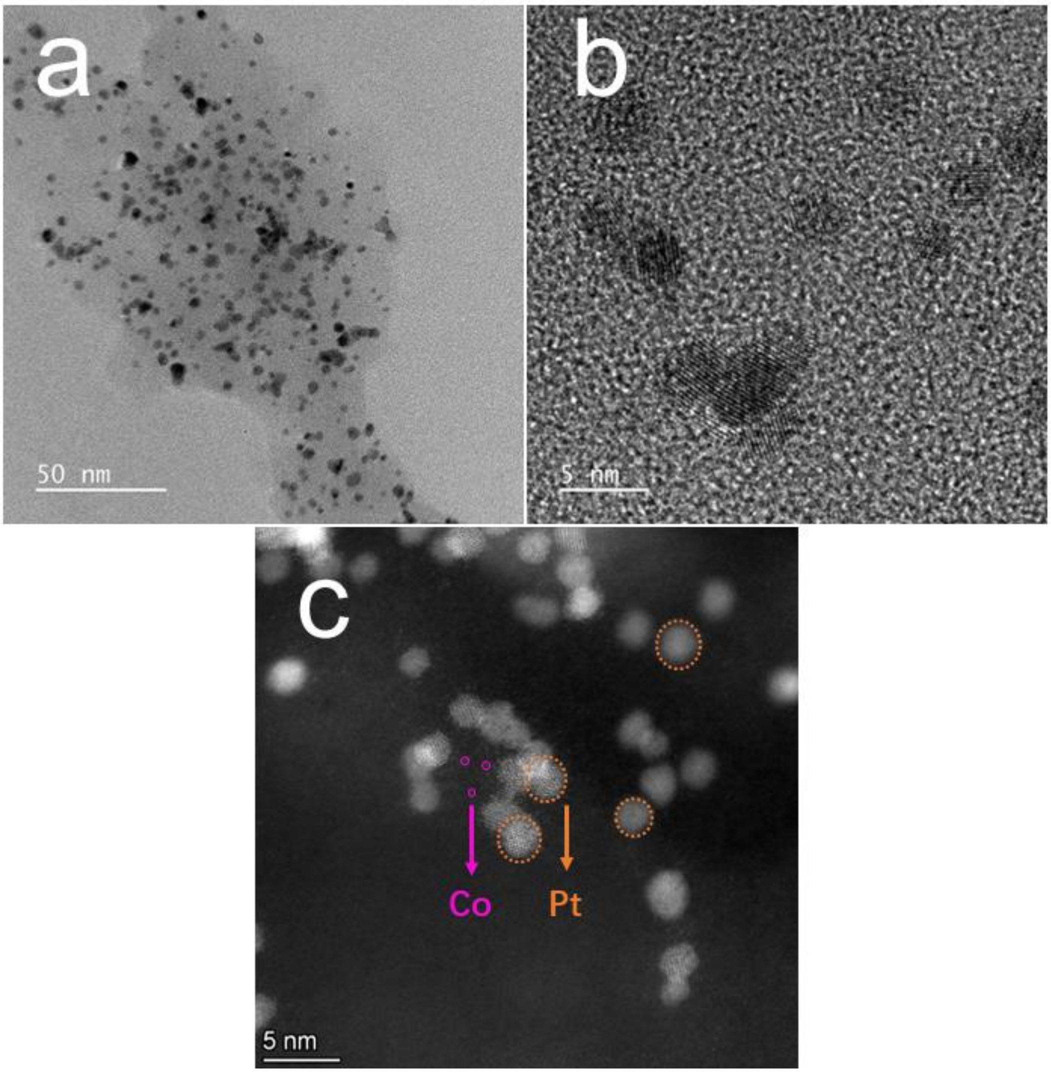

Figure S16 (a), (b) TEM images of PtNC/CPF-Co after stability test. (c) HAADF STEM images of PtNC/CPF-Co after stability test.

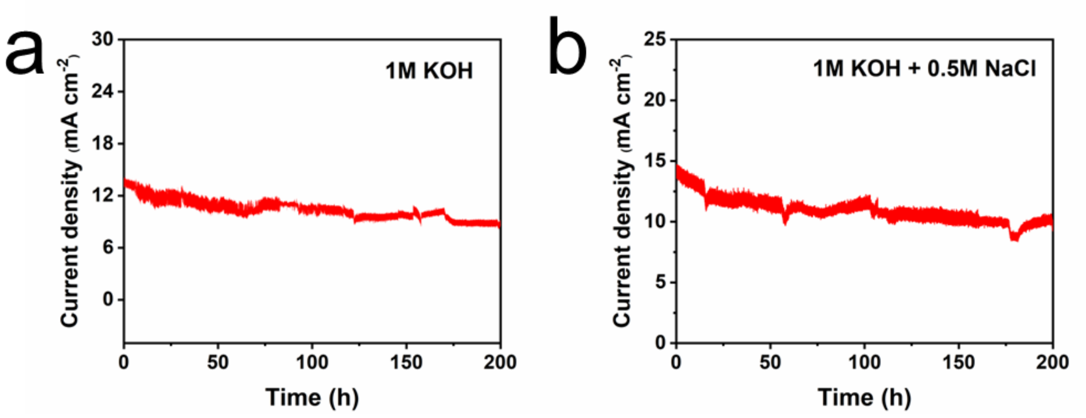

Figure S17 The stability tests of symmetric PtNC/CPF-Co||PtNC/CPF-Co cell in 1 M KOH with and without 0.5 M NaCl.

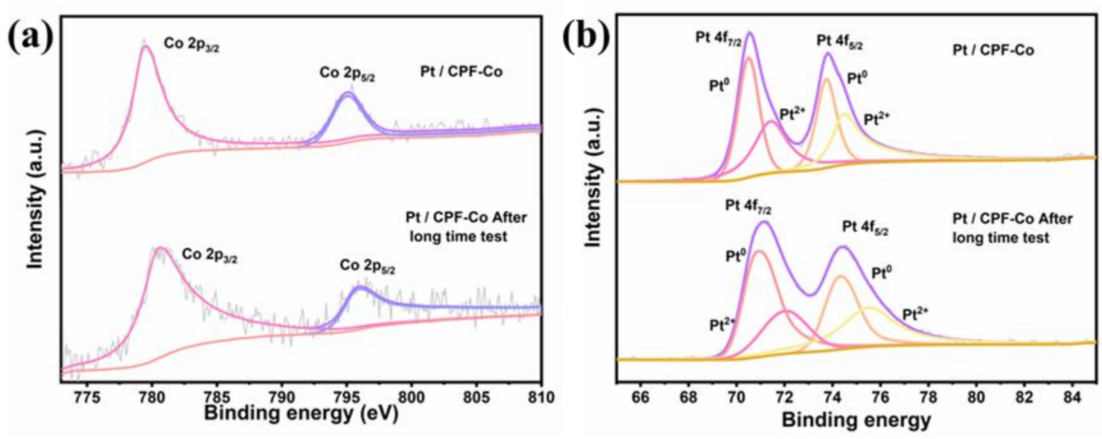

Figure S18. (a) Co 2p spectra and (b) Pt 4f spectra of PtNC/CPF-Co before and after stability test.

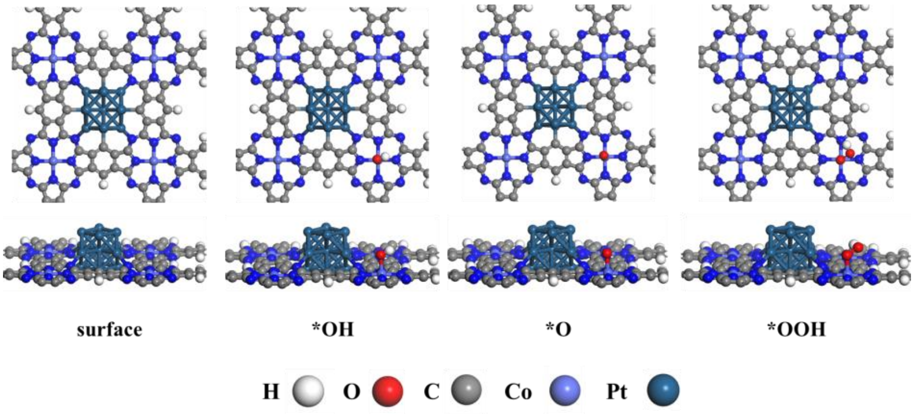

Figure S19 Density functional theory (DFT) calculations for the activity of OER of Co sites on PtNC/CPF-Co.

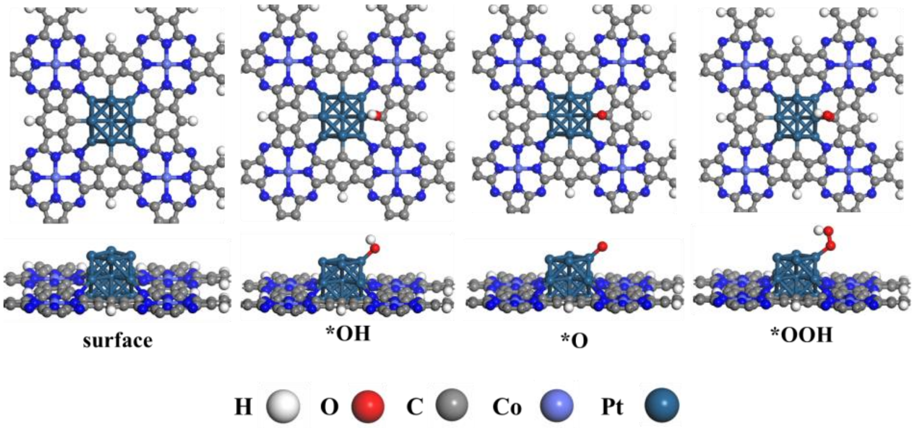

Figure S20 Density functional theory (DFT) calculations for the activity of OER of PtNC sites on PtNC/CPF-Co.

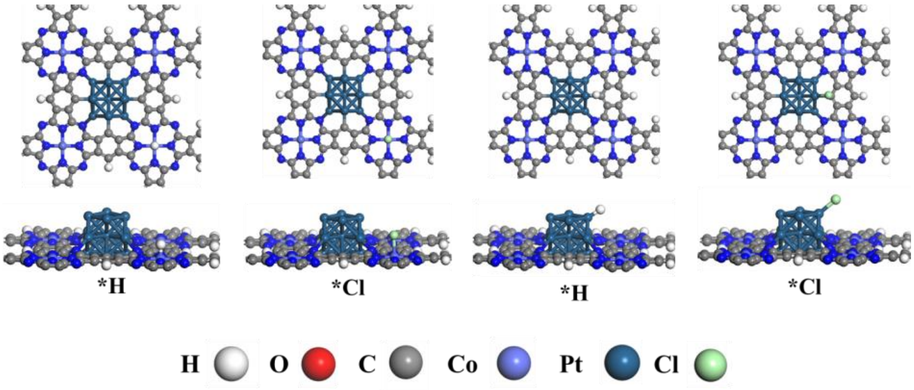

Figure S21 Density functional theory (DFT) calculations for the absorption of H and Cl on the PtNC and Co sites of PtNC/CPF-Co.

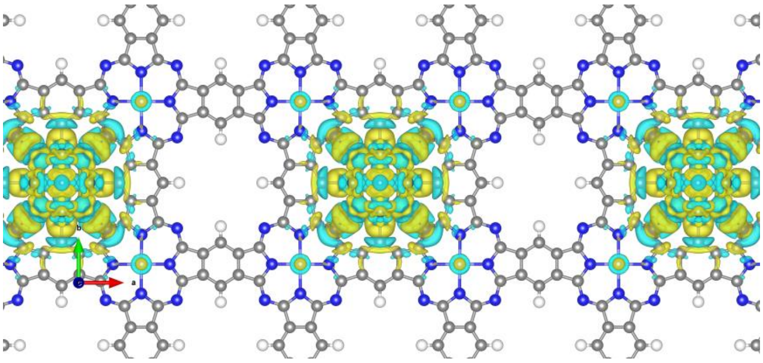

Figure S22 The calculated charge densities of PtNC/CPF-Co (Top View).

Table S1 Fitting parameters for Co K-edge EXAFS of CPF-Co.

Sample	Shell	CN[a]	R(Å)[b]	σ^2(Å^2)[c]	ΔE_0(eV)[d]	R factor
Co foil	Co-Co	12 (set)	2.49	0.007	7.0±0.2	0.003
CPF-Co	Co-N	4.3±0.2	1.88	0.004	0.1±0.5	0.009

[a]*CN*, coordination number; [b]*R*, the distance to the neighboring atom; [c]σ^2, the Mean Square Relative Displacement (MSRD); [d]ΔE_0, inner potential correction; *R* factor indicates the goodness of the fit. $S0^2$ was fixed to 0.7480, according to the experimental EXAFS fit of the sample foil by fixing *CN* as the known crystallographic value. This value was fixed during EXAFS fitting, based on the known structure of Co foil. Data ranges 2.0 ≤ k ≤ 10.0 Å^{-1}, 1.0 ≤ R ≤ 2.0 Å. The Debye-Waller factors and ΔRs are based on the *guessing* parameters and constrained for paths.

Table S2 Fitting parameters for Co K-edge EXAFS of PtNC/CPF-Co.

Sample	Shell	CN[a]	R(Å)[b]	σ^2(Å^2)[c]	ΔE_0(eV)[d]	R factor
Co foil	Co-Co	12 (set)	2.49	0.008	7.1±0.3	0.010
PtNC/CPF-Co	Co-N	4.3±0.3	1.83	0.005	2.9±0.5	0.019

[a]*CN*, coordination number; [b]*R*, the distance to the neighboring atom; [c]σ^2, the Mean Square Relative Displacement (MSRD); [d]ΔE_0, inner potential correction; *R* factor indicates the goodness of the fit. $S0^2$ was fixed to 0.8463, according to the experimental EXAFS fit of the sample foil by fixing *CN* as the known crystallographic value. This value was fixed during EXAFS fitting, based on the known structure of Co foil. Data ranges 2.0 ≤ k ≤ 10.0 Å^{-1}, 1.0 ≤ R ≤ 2.0 Å. The Debye-Waller factors and ΔRs are based on the *guessing* parameters and constrained for paths.

Table S3. The ICP results for PtNC/CPF-Co

Element	Content (wt.%)
Pt	16.50
Co	9.89
